# Competitions between prosocial exclusions and punishments in finite populations

**DOI:** 10.1038/srep46634

**Published:** 2017-04-19

**Authors:** Linjie Liu, Xiaojie Chen, Attila Szolnoki

**Affiliations:** 1 School of Mathematical Sciences, University of Electronic Science and Technology of China, Chengdu 611731, China; 2 Institute of Technical Physics and Materials Science, Centre for Energy Research, Hungarian Academy of Sciences, P.O. Box 49, H-1525 Budapest, Hungary

## Abstract

Prosocial punishment has been proved to be a powerful mean to promote cooperation. Recent studies have found that social exclusion, which indeed can be regarded as a kind of punishment, can also support cooperation. However, if prosocial punishment and exclusion are both present, it is still unclear which strategy is more advantageous to curb free-riders. Here we first study the direct competition between different types of punishment and exclusion. We find that pool (peer) exclusion can always outperform pool (peer) punishment both in the optional and in the compulsory public goods game, no matter whether second-order sanctioning is considered or not. Furthermore, peer exclusion does better than pool exclusion both in the optional and in the compulsory game, but the situation is reversed in the presence of second-order exclusion. Finally, we extend the competition among all possible sanctioning strategies and find that peer exclusion can outperform all other strategies in the absence of second-order exclusion and punishment, while pool exclusion prevails when second-order sanctioning is possible. Our results demonstrate that exclusion is a more powerful strategy than punishment for the resolution of social dilemmas.

Cooperation is widespread in our world, which has a fundamental role on the evolution of human civilization^1^,^2^,^3^,^4^,^5^. However, cooperation is vulnerable to be invaded by selfish individuals who are always maximizing their short-term and immediate interests. Thus how to overcome such individuals is a vital task for the emergence of cooperation in a population^6^,^7^,^8^. Several mechanisms, such as spatial reciprocity, reputation, wisdom of groups, and costly punishment, have been demonstrated to be effective for cooperators to fight against defectors^9^,^10^,^11^,^12^,^13^,^14^,^15^. Staying at the last option, costly punishment has received considerable attention in the last decade because of its importance and widespread prevalence in human societies^16^,^17^,^18^,^19^. By using public goods game (PGG), which is a standard metaphor of social dilemmas, many theoretical and experimental studies have shown that prosocial punishment can reduce the number of free-riders and encourage the majority of individuals to contribute to the common pool^20^,^21^,^22^,^23^.

As an alternative incentive tool to prevent free-riders exploiting community effort, social exclusion can also be observed in human societies^24^,^25^,^26^. It is based on the idea that convicted offenders are denied certain rights and benefits of citizenship or membership of joint ventures^27^. Accordingly, individuals who are identified to violate the rule or jeopardize others’ common interests could be excluded from the community^28^,^29^,^30^,^31^. In this way exclusion serves as a sort of institution to tame defectors not to exploit others. Previous studies have shown that social exclusion can increase social sensitivity^32^,^33^,^34^,^35^,^36^ and induce a positive impact on cooperation when partners are fixed^37^,^38^. Recently, Sasaki and Uchida introduced peer exclusion into the PGG and established a game-theoretical model to study the evolution of social exclusion by using replicator equations in infinite populations^39^. They found that peer exclusion can overcome two shortages of peer punishment: first, a rare punishing cooperator barely subverts the asocial society of free-riders; second, natural selection often eliminates punishing cooperators in the presence of non-punishing cooperators (namely, second-order free-riders). Subsequently, Li *et al*.^40^ studied the comparison between peer exclusion and pool exclusion, and claimed that peer excluders can overcome pool excluders if the exclusion costs are small and excluders can dominate the whole population in a suitable parameters range in the presence of second-order free-riders. Note that pool excluder, similarly to pool punisher, pays a fixed, permanent cost before contributing to the public goods to maintain an institutionalized mechanism for punishing exploiters.

To summarize our present knowledge, both prosocial exclusion and prosocial punishment have been proved to be effective ways for promoting cooperation, but their systematic comparison is still missing. Indeed, the mentioned works^39^,^40^ have compared their independent impacts on the cooperation level, but the consequence of their simultaneous presence is still unexplored. It remained unclear which strategy is more evolutionary advantageous if both exclusion and punishment are simultaneously available for individuals in the population. We wonder if exclusion or punishment is a better way to curb free-riding. How does their relation change if second-order sanctioning is also possible? In the latter case non-punishing individuals or those who deny contribution to the cost of exclusion may also be punished. Furthermore, we also wonder whether peer punishment (peer exclusion) or pool punishment (pool exclusion) is more efficient individual strategy to control transgressors for a higher well-being.

Motivated by these open problems, in this study we focus on the competition between prosocial exclusion and punishment in finite populations who play the PGG. We first investigate the direct competition between pool exclusion and pool punishment, and demonstrate that pool exclusion has the evolutionary advantage over pool punishment both in the optional and in the compulsory PGG, no matter whether second-order exclusion and punishment are considered or not. We then investigate the competition between peer exclusion and peer punishment, and find that peer exclusion is evolutionarily advantageous over peer punishment both in the optional and in the compulsory PGG, independently of the choice of second-order sanctioning. Third, we study the competition between pool exclusion and peer exclusion, and observe that peer exclusion can outperform pool exclusion both in the optional and in the compulsory PGG if second-order exclusion is ignored, while the situation is reversed in the presence of second-order exclusion. Finally, we investigate the full competition of all previously mentioned strategies, such as pool exclusion, peer exclusion, pool punishment, and peer punishment. As our main observation, it turns out that peer exclusion is the most advantageous strategy in the absence of second-order exclusion and punishment, but pool exclusion outperforms other strategies when second-order sanctioning is possible.

## Model

We consider the standard PGG in a finite, well-mixed population with size *M*. In each round of the game, *N* ≥ 2 individuals are selected randomly from the population to form a group for participating in a one-shot game. Then, each individual in the group decides whether or not to contribute an amount of cost *c* to the common pool. The individual who is willing to contribute is called a cooperator, and the individual who does not contribute is called a defector. In the optional PGG we also consider a third option, a strategy which gives up participating in the game, hence is called as a loner. The latter strategy has a constant payoff *σ* which is not affected by others. The sum of the contributions to the common pool is multiplied by the enhancement factor *r* (1 < *r* < *N*), and then equally allocated among all individuals who participated in the game no matter they contributed or not. In agreement with previous works^41^,^42^, if only one individual participates in the game then her income equals with *σ*.

In the second stage of the game exclusion or/and punishment is considered where both related strategies contribute *c* to the common pool. By following refs 21 and 43 peer punishers impose a fine *β* on each free-rider in their group at a cost *γ*. Accordingly, each defector will be fined an amount *βN*_*W*_, where *N*_*W*_ is the number of peer punishers in the group. Pool punishers, however, pay a permanent cost *G* to the punishment pool beforehand. If there exist defectors in the group, they will be fined an amount *BN*_*V*_, where *N*_*V*_ is the number of pool punishers in the group. It simply means that the additional cost of pool punisher is independent of the number of defectors in the group, while the related cost of peer punisher is proportional to the presence of defectors. If considering second-order punishment, second-order free-riders (individuals who contribute to the game but do not bear the extra cost of punishment) will be fined the same amount^21^.

When exclusion is applied we follow conceptually similar protocol as for punishment. Here exclusion serves as a sort of institution to prevent defectors to exploit other group members. Hence the role of excluder can be viewed as a sentinel who alarms other group members about the danger of defectors. Evidently, such an extra effort requires additional cost which is paid by excluder player. Consequently, a peer excluder does not only contribute *c* to the public goods game but also pay a cost *c*_*E*_ after every defector in the group to prevent them collecting benefit from the public goods sharing. In stark contrast to peer exclusion, pool excluders pay a permanent cost *δ* to maintain the institution of exclusion which will block defectors to gain benefit from PGG in the presence of pool excluders. As previously, in case of the second-order exclusion, second-order free-riders (individuals who do not take the extra cost of exclusion) will also be excluded.

In order to study the evolutionary dynamics, we use the so-called pairwise comparison rule with the mutation-selection process^44^,^45^. According to this protocol at each time step a randomly chosen player *i* may change her strategy. We consider the possibility of mutation, hence the player adopts a randomly chosen available strategy with probability *μ*. Alternatively, which happens with probability 1 − *μ*, a player *i* tries to imitate a randomly chosen player *j* with a probability


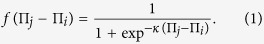


Here Π_*i*_ and Π_*j*_ are the collected payoffs of the mentioned players *i* and *j*, while *κ* characterizes the intensity of selection. In the *κ* → ∞ strong imitation limit the more successful player *j* always succeeds in enforcing her strategy to player *i*, but never otherwise. On the other hand, *κ* → 0 indicates the so-called weak selection limit where strategy adoption becomes random independently of the payoff values. In between these extremes, at a finite value of *κ*, it is likely that a better performing player *j* is imitated, but it is still not impossible to adopt her strategy when performing worse.

In the following we consider four different scenarios when punishment and exclusion compete and we compute the resulting stationary distribution of all available strategies. We suppose a well-mixed finite population where all players interact with each other randomly. To make comparison with previous works easier we have adopted notations for variables by earlier works^21^,^39^. Accordingly, let *X* denote the number of cooperators who contribute to the public pool, but do not bear the cost of punishment or exclusion; *Y* the number of defectors who contribute neither to PGG nor to the sanctions; *Z* the number of loners; *V* the number of pool punishers; *W* the number of peer punishers; *F* the number of pool excluders; and *E* the number of peer excluders. The whole size of population is denoted by *M* and *N* randomly chosen individuals are offered to form a group and establish a joint enterprise. In the next section we present the results of the more complex optional PGG while further details and results for the simplified compulsory PGG game are summarized in the Supplementary Information (SI).

## Results

### Competition between pool exclusion and pool punishment

**Figure 1:**
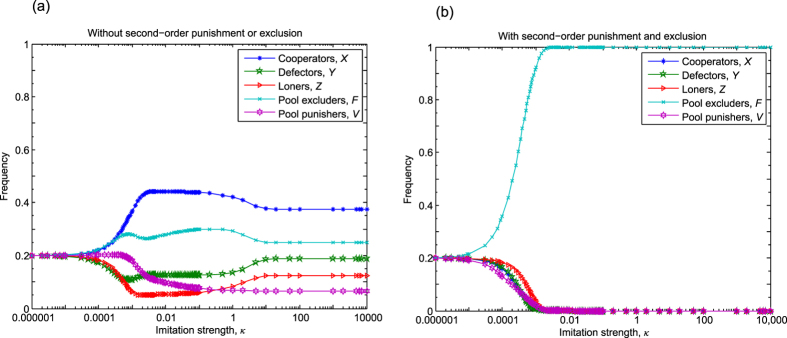
The competition between pool exclusion and pool punishment in the optional PGG. Without second-order exclusion or punishment, shown in panel (a), the frequency of pool excluders is significantly higher than the portion of pool punishers, but all strategies can coexist in time average. In the presence of second-order exclusion and punishment, presented in panel (b), pool excluders prevail and dominate in the strong selection limit. Parameters: *N* = 5, *r* = 3, *c* = 1, *μ* = 10^−6^, *σ* = 1, *M* = 100, *δ* = 0.4, and *G* = *B* = 0.4.

We first study the direct competition between pool exclusion and pool punishment in the optional PGG. In this scenario, there are five available strategies in the population fulfilling the constraint *X* + *Y* + *Z* + *F* + *V* = *M*. We assume that 0 < *σ* < *rc* − *c* − *δ* and 0 < *σ* < *rc* − *c* − *G*, which ensure that a punisher or excluder can get higher profit than a loner if there is more than one participant in the group. In the absence of second-order exclusion and punishment only defectors are sanctioned by punishment or/and exclusion. In Fig. 1(a) we plot the long-run frequencies for each strategy which determine the stationary distribution of all available strategies in dependence of imitation strength *κ*. We find that for *κ* < 10^−4^ the frequencies of the five competing strategies are identical due to the practically random imitation process. As we increase the strength of imitation then all the five strategies can survive and coexist. More precisely, in the perfect imitation limit the system evolves towards a homogeneous state, where the flips between almost homogeneous states are triggered by rare mutations. (A representative trajectory of evolution can be seen in Fig. 1 in ref. 21.) In this way the presented frequencies of stationary states are calculated from the time average of frequencies for competing strategies. As Fig. 1(a) suggests pure cooperators form the highest portion who can enjoy the benefit of exclusion and punishment without paying their costs. Interestingly, the second largest population is formed by pool-excluders followed by defectors and loners, while pool-punishers can make up the smallest fraction, or can be detected with the smallest probability. This result suggests that pool exclusion is more effective against defection and has an evolutionary advantage over pool-punishment strategy. In particular, in the strong imitation (*κ* → ∞) limit the long-run frequencies in the [*X, Y, Z, F, V*] subpopulations are 
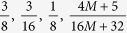
, and 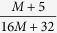, respectively (for further details, see Section 1 in SI).

The comparison of stationary strategies in Fig. 1(a) emphasizes that it is better to cooperate but also to avoid the additional cost of sanctions. Needless to say, if everyone chose this option then we would face the original dilemma. To minimize this undesired consequence of “second-order free-riders” we may penalize those who refuse participating in the sanctioning process. In particular, when second-order exclusion and punishment are considered, we assume that pool-excluders drive out all strategies who do not contribute to the exclusion pool. In parallel, pool-punishers will also punish those who do not bear the additional cost of punishment pool. By applying this scenario, we observe that the long-run frequencies in the [*X, Y, Z, F, V*] subpopulations are [0, 0, 0, 1, 0] for *κ* > 10^−3^, as shown in Fig. 1(b). In other words, when second-order sanctioning is allowed pool-excluders prevail and all other strategies extinct during the evolutionary process. To answer our original question both panels plotted in Fig. 1 highlight that pool-exclusion is a more advantageous strategy than pool-punishment independently of second-order sanctioning is considered or not. We should stress that our observation remains valid for a broad range of parameter interval including the level of the punishment fine or the group size. Furthermore, conceptually identical conclusion can be made if the compulsory PGG is assumed where loner strategy cannot compete (the related results are presented in Sec. 2 of SI).

### Competition between peer exclusion and peer punishment

**Figure 2:**
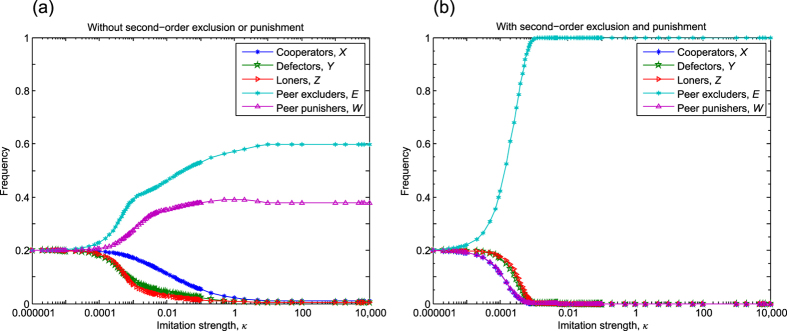
The competition between peer exclusion and peer punishment in the optional PGG. In the absence of second-order sanctioning, shown in panel (a), both strategies survive but peer exclusion dominates. If second-order exclusion and punishment are applied then peer excluders prevail, as shown in panel (b). Both the cost and fine of punishment are equal. Parameters: *N* = 5, *r* = 3, *c* = 1, *μ* = 10^−6^, *σ* = 1, *M* = 100, *c*_*E*_ = 0.4, and *β* = *γ* = 0.4.

In this subsection, we investigate the competition between peer punishment and peer exclusion in the optional PGG. According to this scenario there are also five available strategies in the population whose fractions fulfill *X* + *Y* + *Z* + *E* + *W* = *M*. In the absence of second-order punishment and exclusion, we assume that 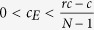, which ensures that a single peer excluder can invade a group of all defectors. In Fig. 2(a) we present the long-run frequencies of the five strategies as a function of the imitation strength *κ*. As expected, for weak selection, when *κ* < 10^−4^, the frequencies of the five strategies are practically identical because of the random strategy updating. By increasing the imitation strength *κ* peer excluder strategy becomes gradually dominant and occupies the majority of the population. Peer punisher strategy can only reach the second best position in the rank of strategies. In the strong imitation limit the long-run frequencies for the [*X, Y, Z, E, W*] subpopulations are 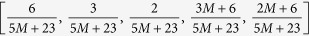 (see Section. 3 of SI). This suggests that the number of peer excluders is about 1.5 times larger in time average than the second best peer punishers for large population size, hence demonstrating the superiority of the former strategy.

If second-order sanctioning is allowed then the relation of sanctioning strategies becomes even more unambiguous. Interestingly, in this case excluders do not only ostracize pure cooperators but also punishers who refuse to contribute to the cost of exclusion. But the penalty works also in the reversed direction because punishers lower the payoff of both cooperation and exclusion strategies. The consequence of this mutual sanctioning is summarized in Fig. 2(b), which suggests that peer exclusion prevails and gives no space for any other strategies. This observation supports our previous conclusion about the effectiveness of exclusion that is not restricted to pool strategies, but is still valid for peer strategies. We stress that this conclusion remains unchanged if we release the restriction for the value of *c*_*E*_, which means that for 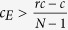 peer exclusion has still evolutionary advantage over peer punishment. The border within this observation is valid can be extended further because peer exclusion outperforms peer punishment in the compulsory PGG, no matter whether second-order sanctioning is applied or not (for more details see Section 4 in SI).

### Competition between pool exclusion and peer exclusion

**Figure 3:**
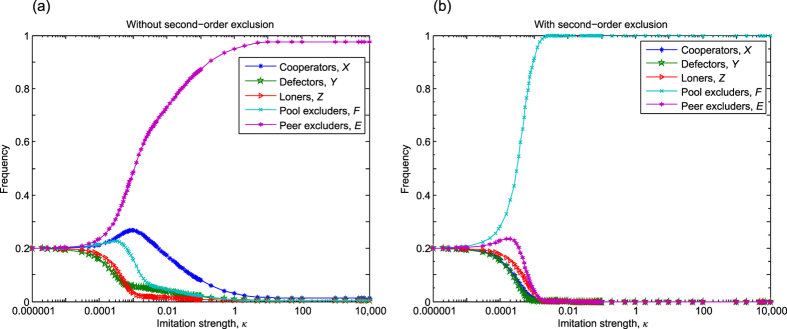
The competition between pool exclusion and peer exclusion in the optional PGG. In the absence of second-order exclusion, peer excluders prevail, shown in panel (a). Panel (b) shows the opposite case which happens if second-order exclusion is applied. Parameters: *N* = 5, *r* = 3, *c* = 1, *μ* = 10^−6^, *σ* = 1, *M* = 100, and *c*_*E*_ = *δ* = 0.4.

In the following subsection we compare the peer- and pool-exclusion strategies in the optional PGG, which are proved to be more effective than their punishing mates in the previously studied cases. Here, there are five available strategies in the population whose fractions fulfill the constraint *X* + *Y* + *Z* + *F* + *E* = *M*. For their proper comparison we assume that their costs remain below the previously established limit, that is 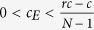 and 0 < *σ* < *rc* − *c* − *δ*. First, we consider the case when second-order exclusion is not allowed, hence both excluder strategies penalize pure defectors only. Figure 3(a) illustrates that peer excluder strategy becomes dominant as we gradually increase the imitation strength. All the other strategies can share a reasonable portion only at an intermediate value *κ*. If the imitation strength exceeds the threshold *κ* > 10^−1^ then the long-run frequencies of defectors, loners, and pool excluders are close to zero, and only cooperators can coexist with peer excluders. In particular, the fractions of [*X, Y, Z, F, E*] strategies in the strong imitation (*κ* → ∞) limit are 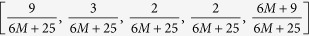 (further details can be seen in Section 5 of SI). These results suggest that peer excluder strategy is able to dominate the whole population in the absence of second-order exclusion.

Interestingly, the outcome of evolutionary trajectory is completely reversed if second-order exclusion is considered. In this case, in strong agreement with a previous work where peer- and pool-punisher strategies were compared^21^, pool excluders are capable to crowd out peer excluders. The result of this competition is summarized in Fig. 3(b) where the long-run frequencies for each strategy are plotted. In the strong imitation limit the victory of pool excluders is total, yielding [0, 0, 0, 1, 0] fractions for *X, Y, Z, F*, and *E* strategies respectively. As in the previous cases, these results remain valid if the compulsory PGG is played. Here, in the absence of loners, peer-excluders dominate when second-order exclusion is not considered yielding 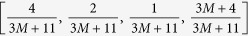 values for the competing *X, Y, F*, and *E* strategies in the strong imitation limit (details can be found in Sec. 6 of SI). When second-order exclusion is possible then the pool excluder strategy prevails in close agreement with the result of optional PGG.

### Competition between prosocial exclusions and punishments

**Figure 4:**
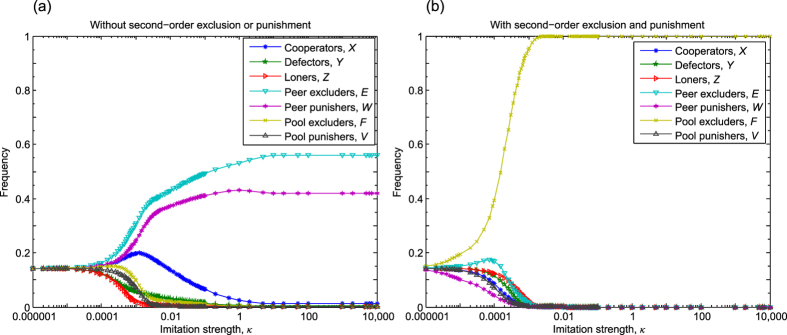
The competition between different types of social exclusion and costly punishment in the optional PGG. When strategies can penalize defectors only, shown in panel (a), then all strategies coexist in time average for weak strength of imitation, but in most of the time peer excluders form the majority of the population for other imitation strength values. Panel (b) shows the case when second-order sanctioning is possible. Here pool excluders prevail and conquer the whole population. Parameters: *N* = 5, *r* = 3, *c* = 1, *μ* = 10^−6^, *σ* = 1, *M* = 100, *c*_*E*_ = *δ* = 0.4, *β* = *γ* = 0.4, and *B* = *G* = 0.4.

The pair comparison of competing strategies may provide a first guide about their relations, but the presence of a third party could be a decisive factor, which may completely rearrange the ranks of competitors. To clarify this possibility in the following we explore the simultaneous competitions of all previously studied strategies. Namely, we consider an optional PGG where seven strategies, namely pure cooperator, defector, loner, peer excluder, peer punisher, pool excluder, and pool punisher are present. As in the previous cases, we first consider the option when second-order sanctions are not applied hence only defectors suffer from the presence of excluders and punishers. Figure 4(a) summarizes our results, which suggest that “peer-sactioning” strategies are the most effective, but more importantly, peer excluders can dominate the population. In this way the dominance of peer excluders over peer punishers is not disturbed by the presence of other sanctioning strategies such as pool excluders or pool punishers. As Fig. 4(a) shows all the other strategies become irrelevant in the strong imitation limit. In particular, the long-run frequencies of *X, Y, Z, E, W, F* and *V* subpopulations are 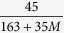, 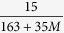, 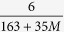, 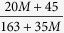, 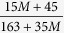, 
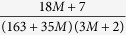
, and 
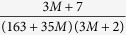
, respectively (further details can be seen in Section 7 of SI). This result suggests that only the sanctioning strategies survive in the large population limit where the majority of individuals are peer excluders in most of the time.

In the next logical step we consider the case when second-order sanctioning is possible. This option offers an extremely complex food-web between competing strategies, because practically all sanctioning strategies try to beat all the others. For instance, pool excluders ostracize not only defectors and simple cooperators, but also peer excluders, peer punishers, and pool punishers. In this “almost everybody beats everybody else” battle the final victor is pool excluder strategy. This case is plotted in Fig. 4(b) where the resulting fractions of the strategies are [0, 0, 0, 0, 0, 1, 0] in the strong imitation limit (further details can be seen in Sec. 7 of SI).

To close this section we briefly summarize the results of the compulsory PGG where 6 competing strategies remain. The details of the calculation can be found in Sec. 8 of SI. In the absence of second-order exclusion and punishment, we find that the behaviour is conceptually similar to the one we observed for the optional PGG. Here peer excluders and peer punishers perform the best, but all the other strategies survive at intermediate strength of imitation. In the strong imitation limit the resulting fractions of *X, Y, E, W, F*, and *V* strategies are 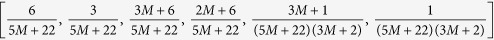, which suggests that only sanctioning strategies survive in the large-population limit. When second-order exclusion and punishment are possible then we get back the result obtained previously for the optional PGG: only pool excluders survive for strong enough imitation strength.

## Discussion

Penalizing free-riders whose behaviour threaten the collective efforts seems almost inevitable. But which sanctioning tool shall we apply to reach our goal efficiently? To punish them by lowering their payoffs or to deny their rights to enjoy the benefit of public goods? The answer could be even more complicated because both peer and pool sanctioning can be used. While peer punishers and peer excluders invest an extra cost only in the presence of defectors, pool punisher and pool excluder strategies apply a permanent effort to maintain the sanctioning institutions. Based on previous works both punishment and exclusion seem to be appropriate methods^21^,^46^, but their systematic comparison has not been done yet.

In this work, we have thus studied the competitions between costly punishments and exclusions in finite populations playing the PGG by using different scenarios. For a fair comparison we have applied equally high cost of punishment and exclusion. We have found that peer exclusion is always favored by natural selection when it competes with peer punishment both in the optional and in the compulsory PGG, independently of second-order punishment and exclusion are considered or not. Conceptually similar findings have been obtained for pool exclusion when it directly competes with pool punishment. Furthermore, when peer exclusion competes with pool exclusion, peer exclusion wins in the absence of second-order exclusion, while pool exclusion prevails when second-order exclusion is applied. Lastly, we have also explored the most complex option when all four sanctioning methods compete with the pure cooperator, defector, and loner strategies. In the latter case peer exclusion is proved to be the most viable tool in the absence of second-order punishment and exclusion, while pool exclusion prevails when second-order sanctioning is allowed. To sum up, the systematic comparison of sanctioning strategies highlights that exclusion is always a more effective way to control free-riders than punishment, but the absence or the presence of second-order sanctioning could be a decisive factor, because the former condition supports peer exclusion while the latter option helps pool exclusion strategy to prevail.

We would like to stress that our finding is robust and remains valid in a broad range of model parameters (some representative plots are given in Sec. 9 of SI). For instance, if we increase the punishment fine by fixing the cost of punishment then the superiority of exclusion is still not in danger. In general, if the fine is not unrealistically high and the cost of exclusion does not exceed the cost of punishment then exclusion strategy always performs better similarly to the cases we discussed earlier. Indeed, we have verified that in the absence of second-order sanctioning the exclusion strategy still has an evolutionary advantage over the punishment strategy, no matter an enhanced fine value applied by peer and pool punishers. To give an example, the outcomes remain conceptually intact when the punishment fine exceeds eight times the punishment cost. But if second-order sanctioning is applied at such severe punishment then the advantage of excluders diminishes because their payoff becomes negative, which implies the victory of punishers. However, we should note that applying such a severe punishment is not an attractive feature when humans qualify potential social partners^47^. We have also considered different group sizes and found that it has no significant role in the competition of sanctioning strategies (this is demonstrated clearly in Fig. S7 of SI).

In order to provide a convenient framework for studying the competitions between costly exclusions and punishments, we focused on the option when free-riders are always exiled in the presence of excluders who have to bear the related cost. A further step could be when this sanction is not perfect and exclusion happens in a probabilistic manner^39^,^40^. Indeed, previous works emphasized the value of probabilistic sanctioning^19^,^48^, which opens promising avenue for future studies. Our work can be also extended where the error of perception, i.e. defectors are identified with some ambiguity, or the error of punishment or exclusion are also considered. In the latter cases innocent players are punished or excluded from the joint venture by mistake. To consider anti-social punishment and anti-social exclusion may also open interesting research avenue to explore the effectiveness of exclusion^49^,^50^,^51^. Lastly, we note that our calculation is restricted to the simplest, well-mixed population because of the extremely high number of competing strategies. However, it is a frequently discussed fact that in structured populations, where interaction topology is considered, the evolutionary outcomes could be significantly different from those presented for mean-field systems^52^,^53^,^54^,^55^,^56^,^57^. Therefore, we expect similar exciting new observations from related efforts which will hopefully make our understanding more accurate.

## Additional Information

**How to cite this article**: Liu, L. *et al*. Competitions between prosocial exclusions and punishments in finite populations. *Sci. Rep.* **7**, 46634; doi: 10.1038/srep46634 (2017).

**Publisher's note:** Springer Nature remains neutral with regard to jurisdictional claims in published maps and institutional affiliations.

## Supplementary Material

Supplementary Information
