# Supplementary material for: Competitions between prosocial exclusions and punishments in finite populations
Source: Sci Rep. 2017 Apr 19;7:46634. doi: 10.1038/srep46634 (PMC5395949; doi:10.1038/srep46634)
Supplement: Supplementary Information [file srep46634-s1.pdf]

Supplementary Information: Competitions between prosocial exclusions and punishments in finite populations

Linjie Liu,[1] Xiaojie Chen,[1] and Attila Szolnoki[2]

[1]School of Mathematical Sciences, University of Electronic Science and Technology of China, Chengdu 611731, China

[2]Institute of Technical Physics and Materials Science, Centre for Energy Research, Hungarian Academy of Sciences, P.O. Box 49, H-1525 Budapest, Hungary

In the Supplementary Information (SI), we provide the specific analysis for the strategy distribution in the finite population with size M by elaborating eight different scenarios. In order to calculate the stationary distribution of more than two strategies in our study, we consider that the mutation probability μ is sufficiently small that is in agreement with previous works [1–4]. Consequently, the population will never contain more than two different strategies simultaneously, and the population can evolve into a homogeneous state where all individuals adopt the same strategy because the time between two mutation events is long enough. This assumption allows us to approximate the evolutionary dynamics by means of an embedded Markov chain whose states correspond to the different homogeneous states of the population. If the number of available strategies is denoted by d then the state transition matrix which describes the different transition probabilities for the population to move from one state to the other is given by

$$\mathbf{A} = [a_{hq}]_{d\times d},$$

where a_{hq} is the probability that the system switches from the h state (where all individuals adopt strategy h) to the q state (where all individuals adopt strategy q) after the emergence of a single mutation). Here $a_{hq} = \frac{\rho_{hq}}{d-1}$ if $h \neq q$ and $a_{hq} = 1 - \sum_{q\neq h} \frac{\rho_{hq}}{d-1}$ otherwise, where ρ_{hq} is the fixation probability that a single individual with strategy q takes over a resident population of individuals with strategy h. The stationary distribution of the population can be calculated from the average fraction of time that population spends in each of the homogeneous states. Technically, it is given by the normalized left eigenvector of the eigenvalue 1 of the transition matrix $\mathbf{A}$.

The fixation probability ρ_{hq} can be calculated as follows. We assume that in a finite population with size M there are H individuals using strategy h and $Q = M - H$ individuals using strategy q. Then the probability that the number of individuals who use strategy h increases/decreases by one is

$$T^{\pm}(H) = \frac{H}{M}\frac{M-H}{M}\frac{1}{1+exp^{\mp\kappa(\Pi_{hq}-\Pi_{qh})}},$$

where Π_{hq} and Π_{qh} are the average payoffs of individuals with strategy h and with strategy q, depending on the numbers H and Q. Correspondingly, the fixation probability ρ_{hq} can be expressed by

$$\begin{aligned}\rho_{hq} &= \frac{1}{1+\sum_{m=1}^{M-1}\prod_{Q=1}^{m}\frac{T^-(H)}{T^+(H)}} \\ &= \frac{1}{1+\sum_{m=1}^{M-1} exp^{[\kappa\sum_{Q=1}^{m}(\Pi_{hq}-\Pi_{qh})]}}.\end{aligned}$$

1 Competition between pool exclusion and pool punishment in the optional PGG

First, we calculate the average payoffs for each strategy in a finite population assuming that M individuals consist of X cooperators, Y defectors, Z loners, F pool excluders, and V pool punishers. Thus the probability to find no pool excluders in the population is

$$\frac{\binom{M-F-1}{N-1}}{\binom{M-1}{N-1}}.$$

Accordingly, the average payoff for cooperators is given by

$$\begin{aligned}\Pi_X = \frac{\binom{Z}{N-1}}{\binom{M-1}{N-1}}\sigma + [1 - \frac{\binom{Z}{N-1}}{\binom{M-1}{N-1}}]\{[1 - \frac{\binom{M-F-1}{N-1}}{\binom{M-1}{N-1}}](rc - c) \\ + \frac{\binom{M-F-1}{N-1}}{\binom{M-1}{N-1}} \sum_{k=0}^{N-1} \sum_{j=0}^{N-k-1} \frac{\binom{M-Y-Z-F-1}{N-j-k-1}\binom{Y}{j}\binom{Z}{k}}{\binom{M-F-1}{N-1}} [\frac{r(N-j-k)c}{N-k} - c]\},\end{aligned}$$

where k represents the number of loners, and j represents the number of defectors in the group. Defectors are not only excluded by pool excluders, but also punished by pool punishers. Thus, the average payoff for defectors is

$$\begin{aligned}\Pi_Y = \frac{\binom{Z}{N-1}}{\binom{M-1}{N-1}}\sigma + [1 - \frac{\binom{Z}{N-1}}{\binom{M-1}{N-1}}]\{[1 - \frac{\binom{M-F-1}{N-1}}{\binom{M-1}{N-1}}][-\frac{(N-1)VB}{M-1}] + \frac{\binom{M-F-1}{N-1}}{\binom{M-1}{N-1}} \\ \sum_{i=0}^{N-1} \sum_{j=0}^{N-i-1} \sum_{k=0}^{N-i-j-1} \frac{\binom{M-Y-X-Z-F}{N-i-j-k-1}\binom{Y-1}{j}\binom{Z}{k}\binom{X}{i}}{\binom{M-F-1}{N-1}} [\frac{r(N-j-k-1)c}{N-k} - (N-i-j-k-1)B]\},\end{aligned}$$

where i represents the number of cooperators. The average payoff for loners is

$$\Pi_Z = \sigma.$$

The average payoff for pool excluders is

$$\Pi_F = \frac{\binom{Z}{N-1}}{\binom{M-1}{N-1}}\sigma + [1 - \frac{\binom{Z}{N-1}}{\binom{M-1}{N-1}}](rc - c - \delta).$$

Finally, the pool punishers' payoff is

$$\begin{aligned}\Pi_V = \frac{\binom{Z}{N-1}}{\binom{M-1}{N-1}}\sigma + [1 - \frac{\binom{Z}{N-1}}{\binom{M-1}{N-1}}]\{[1 - \frac{\binom{M-F-1}{N-1}}{\binom{M-1}{N-1}}](rc - c - G) \\ + \frac{\binom{M-F-1}{N-1}}{\binom{M-1}{N-1}} \sum_{j=0}^{N-1} \sum_{k=0}^{N-j-1} \frac{\binom{M-F-Y-Z-1}{N-j-k-1}\binom{Y}{j}\binom{Z}{k}}{\binom{M-F-1}{N-1}} [\frac{r(N-j-k)c}{N-k} - c - G]\}.\end{aligned}$$

In what follows, we calculate the elements of the transition matrix $\mathbf{A}$ to determine the stationary distribution of the population. First, we calculate the payoff expressions Π_{hq} of strategy type h competing against type q for all the possible pairs, which are the followings.

$$
\begin{aligned}
\Pi_{XY} &= \sum_{i=0}^{N-1} \frac{\binom{X-1}{i}\binom{M-X}{N-i-1}}{\binom{M-1}{N-1}}[\frac{r(i+1)c}{N} - c] = \frac{rc}{N}[\frac{(N-1)(X-1)}{M-1} + 1] - c, \\
\Pi_{YX} &= \sum_{i=0}^{N-1} \frac{\binom{X}{i}\binom{M-X-1}{N-i-1}}{\binom{M-1}{N-1}}\frac{ric}{N} = \frac{rc}{N}\frac{(N-1)X}{M-1}, \\
\Pi_{XZ} &= \frac{\binom{Z}{N-1}}{\binom{M-1}{N-1}}\sigma + [1 - \frac{\binom{Z}{N-1}}{\binom{M-1}{N-1}}](rc-c) = rc - c - \frac{\binom{Z}{N-1}}{\binom{M-1}{N-1}}(rc-c-\sigma), \\
\Pi_{ZX} &= \Pi_{ZY} = \Pi_{ZF} = \Pi_{ZV} = \sigma, \\
\Pi_{XF} &= \Pi_{XV} = rc - c, \\
\Pi_{FX} &= \Pi_{FY} = \Pi_{FV} = rc - c - \delta, \\
\Pi_{VX} &= \Pi_{VF} = rc - c - G, \\
\Pi_{YF} &= 0, \\
\Pi_{YZ} &= \frac{\binom{Z}{N-1}}{\binom{M-1}{N-1}}\sigma, \\
\Pi_{YV} &= \frac{(N-1)V}{M-1}(\frac{rc}{N} - B), \\
\Pi_{FZ} &= rc - c - \delta - \frac{\binom{Z}{N-1}}{\binom{M-1}{N-1}}(rc-c-\delta-\sigma), \\
\Pi_{VY} &= \frac{rc}{N}[\frac{(N-1)(V-1)}{M-1} + 1] - c - G, \\
\Pi_{VZ} &= rc - c - G - \frac{\binom{Z}{N-1}}{\binom{M-1}{N-1}}(rc-c-G-\sigma).
\end{aligned}
$$

Based on the above payoff expressions, we can give the embedded Markov chain describing the transition between cooperators (X), defectors (Y), loners (Z), pool excluders (F), and pool excluders (V) as

$$
\begin{pmatrix}
I_X & \frac{\rho_{XY}}{4} & \frac{\rho_{XZ}}{4} & \frac{\rho_{XF}}{4} & \frac{\rho_{XV}}{4} \\
\frac{\rho_{YX}}{4} & I_Y & \frac{\rho_{YZ}}{4} & \frac{\rho_{YF}}{4} & \frac{\rho_{YV}}{4} \\
\frac{\rho_{ZX}}{4} & \frac{\rho_{ZY}}{4} & I_Z & \frac{\rho_{ZF}}{4} & \frac{\rho_{ZV}}{4} \\
\frac{\rho_{FX}}{4} & \frac{\rho_{FY}}{4} & \frac{\rho_{FZ}}{4} & I_F & \frac{\rho_{FV}}{4} \\
\frac{\rho_{VX}}{4} & \frac{\rho_{VY}}{4} & \frac{\rho_{VZ}}{4} & \frac{\rho_{VF}}{4} & I_V
\end{pmatrix},
$$

where $I_K = 1 - \sum_{K \neq L} \frac{\rho_{KL}}{4}$, and $K, L \in \{X, Y, Z, F, V\}$. Correspondingly, we can determine the long-run frequency for each strategy as a function of the imitation strength κ [see Fig. 1(a)].

In the strong imitation limit the embedded Markov chain describing the transitions matrix is simplified as

$$\begin{pmatrix} \frac{3}{4} & \frac{1}{4} & 0 & 0 & 0 \\ 0 & \frac{1}{2} & \frac{1}{4} & \frac{1}{4} & 0 \\ \frac{1}{8} & 0 & \frac{5}{8} & \frac{1}{8} & \frac{1}{8} \\ \frac{1}{4} & 0 & 0 & \frac{3}{4}-\frac{1}{4M} & \frac{1}{4M} \\ \frac{1}{4} & 0 & 0 & \frac{1}{4M} & \frac{3}{4}-\frac{1}{4M} \end{pmatrix}.$$

Accordingly, the stationary distribution is $[\frac{3}{8}, \frac{3}{16}, \frac{1}{8}, \frac{4M+5}{16M+32}, \frac{M+5}{16M+32}]$, which demonstrates that all the five strategists can coexist, but the frequency of pool punishers is significantly lower than pool excluders.

In the presence of second-order exclusion and punishment, we assume that pool excluders exclude pool punishers, pure cooperators, and defectors. And pool punishers punish pool excluders, pure cooperators, and defectors. The average payoffs for defectors and loners are not changed. But the average payoff for cooperators is given by

$$\Pi_X = \frac{\binom{Z}{N-1}}{\binom{M-1}{N-1}}\sigma + [1-\frac{\binom{Z}{N-1}}{\binom{M-1}{N-1}}]\{[1-\frac{\binom{M-F-1}{N-1}}{\binom{M-1}{N-1}}][-c-\frac{(N-1)VB}{M-1}]+ \frac{\binom{M-F-1}{N-1}}{\binom{M-1}{N-1}}\sum_{i=0}^{N-1}\sum_{k=0}^{N-1-i}\sum_{p=0}^{N-1-i-k}\frac{\binom{M-X-Y-F-Z}{p}\binom{Z}{k}\binom{X-1}{i}\binom{Y}{N-i-k-p-1}}{\binom{M-F-1}{N-1}}[\frac{rc(i+p+1)}{N-k}-c-pB]\},$$

where p represents the number of pool punishers in the group. The average payoff for pool excluders is given by

$$\Pi_F = \frac{\binom{Z}{N-1}}{\binom{M-1}{N-1}}\sigma + [1-\frac{\binom{Z}{N-1}}{\binom{M-1}{N-1}}]\sum_{i=0}^{N-1}\sum_{k=0}^{N-1-i}\sum_{p=0}^{N-1-i-k}\frac{\binom{M-X-Y-V-Z-1}{N-i-k-p-1}\binom{Z+Y}{k}\binom{X}{i}\binom{V}{p}}{\binom{M-1}{N-1}}[\frac{rc(N-k)}{N-i-p-k} -c-\delta-pB].$$

Last, the payoff for pool punishers is

$$\Pi_V = \frac{\binom{Z}{N-1}}{\binom{M-1}{N-1}}\sigma + [1-\frac{\binom{Z}{N-1}}{\binom{M-1}{N-1}}]\{[1-\frac{\binom{M-F-1}{N-1}}{\binom{M-1}{N-1}}](-c-G)+ \frac{\binom{M-F-1}{N-1}}{\binom{M-1}{N-1}}\sum_{i=0}^{N-1}\sum_{k=0}^{N-1-i}\sum_{p=0}^{N-1-i-k}\frac{\binom{M-X-Y-F-Z-1}{p}\binom{Z}{k}\binom{X}{i}\binom{Y}{N-i-k-p-1}}{\binom{M-F-1}{N-1}}[\frac{rc(i+p+1)}{N-k}-c-G]\}.$$

Again, the transition matrix between cooperators (X), defectors (Y), loners (Z), pool excluders

(F), and pool punishers (V) can be simplified in the strong imitation limit as

$$\begin{pmatrix} \frac{1}{2} & \frac{1}{4} & 0 & \frac{1}{4} & 0 \\ 0 & \frac{1}{2} & \frac{1}{4} & \frac{1}{4} & 0 \\ \frac{1}{8} & 0 & \frac{5}{8} & \frac{1}{8} & \frac{1}{8} \\ 0 & 0 & 0 & 1 & 0 \\ 0 & 0 & 0 & \frac{1}{4} & \frac{3}{4} \end{pmatrix}.$$

The resulting stationary distribution is $[0, 0, 0, 1, 0]$, which suggests that pool excluders prevail [results are summarized in Fig. 1(b)].

2 Competition between pool exclusion and pool punishment in the compulsory PGG

In this section we assume that the population contains X cooperators, Y defectors, F pool excluders, and V pool punishers from which N individuals are selected randomly to play the PGG. In the absence of second-order exclusion and punishment, a cooperator obtains the average payoff as

$$\begin{aligned}\Pi_X &= [1 - \frac{\binom{M-F-1}{N-1}}{\binom{M-1}{N-1}}](rc - c) + \frac{\binom{M-F-1}{N-1}}{\binom{M-1}{N-1}} \sum_{i=0}^{N-1} \frac{\binom{M-Y-F-1}{i}\binom{Y}{N-i-1}}{\binom{M-F-1}{N-1}} [\frac{r(i+1)c}{N} - c] \\ &= rc[1 - \frac{\binom{M-F-1}{N-1}}{\binom{M-1}{N-1}} \frac{(N-1)Y}{N(M-F-1)}] - c.\end{aligned}$$

The payoff for defectors is

$$\begin{aligned}\Pi_Y \;&=\; [1 - \frac{\binom{M-F-1}{N-1}}{\binom{M-1}{N-1}}] \sum_{p=0}^{N-1} \frac{\binom{M-V-1}{N-p-1}\binom{V}{p}}{\binom{M-1}{N-1}} (-pB) + \\ &\quad \frac{\binom{M-F-1}{N-1}}{\binom{M-1}{N-1}} \sum_{j=0}^{N-1} \sum_{p=0}^{N-1-j} \frac{\binom{M-Y-F-V}{N-j-p-1}\binom{V}{p}\binom{Y-1}{j}}{\binom{M-F-1}{N-1}} [\frac{r(N-j-1)c}{N} - pB] \\ &= -\frac{\binom{M-F-1}{N-1}}{\binom{M-1}{N-1}} \frac{(N-1)[FVNB - (M-F-Y)rc(M-1)]}{N(M-F-1)(M-1)} - \frac{(N-1)VB}{M-1}.\end{aligned}$$

The payoff for pool excluders is

$$\Pi_F = rc - c - \delta.$$

Finally, the payoff for pool punishers is

$$\Pi_V = rc - c - G - \frac{\binom{M-F-1}{N-1}}{\binom{M-1}{N-1}} \frac{rc(N-1)Y}{N(M-F-1)}.$$

For small mutation rate, the embedded Markov chain describing the transitions between cooperators (X), defectors (Y), pool excluders (F), and pool punishers (V) is given by

$$\begin{pmatrix} I_X & \frac{\rho_{XY}}{3} & \frac{\rho_{XF}}{3} & \frac{\rho_{XV}}{3} \\ \frac{\rho_{YX}}{3} & I_Y & \frac{\rho_{YF}}{3} & \frac{\rho_{YV}}{3} \\ \frac{\rho_{FX}}{3} & \frac{\rho_{FY}}{3} & I_F & \frac{\rho_{FV}}{3} \\ \frac{\rho_{VX}}{3} & \frac{\rho_{VY}}{3} & \frac{\rho_{VF}}{3} & I_V \end{pmatrix},$$

where $I_K = 1 - \sum_{K \neq L} \frac{\rho_{KL}}{3}$, and $K, L \in \{X, Y, F, V\}$.

For strong imitation, we set that $\delta = G$, then the transition matrix is given by

$$\begin{pmatrix} \frac{2}{3} & \frac{1}{3} & 0 & 0 \\ 0 & \frac{2}{3} & \frac{1}{3} & 0 \\ \frac{1}{3} & 0 & \frac{2}{3} - \frac{1}{3M} & \frac{1}{3M} \\ \frac{1}{3} & 0 & \frac{1}{3M} & \frac{2}{3} - \frac{1}{3M} \end{pmatrix}.$$

In this case the resulting stationary distribution is $[\frac{1}{3}, \frac{1}{3}, \frac{M+1}{3M+6}, \frac{1}{3M+6}]$. It suggests that pool punishers become extinct for large population size, and the surviving three strategies beat each other by forming a Rock-Paper-Scissors-like cyclic dominance [see Fig. S1(a)].

In the presence of second-order exclusion and punishment, we assume that pool excluders exclude pool punishers, pure cooperators, and defectors. Similarly, pool punishers punish pool excluders, pure cooperators, and defectors. While the payoff for defectors remains unchanged, the average payoff for cooperators is modified by

$$\Pi_X = -c - \frac{(N-1)VB}{M-1} + \frac{\binom{M-F-1}{N-1}}{\binom{M-1}{N-1}} \{ \frac{-NFVB(N-1) + rc(M-1)[N(M-F-1) - (N-1)Y]}{N(M-1)(M-F-1)} \}.$$

The payoff for pool excluders is

$$\Pi_F = \sum_{l=0}^{N-1} \sum_{i=0}^{N-l-1} \sum_{p=0}^{N-i-l-1} \frac{\binom{M-X-Y-V-1}{l}\binom{X}{i}\binom{V}{p}\binom{Y}{N-i-p-l-1}}{\binom{M-1}{N-1}} [\frac{r(i+l+p+1)c}{l+1} - c - pB - \delta],$$

where l represents the number of pool excluders in the group. Last, the payoff for pool punishers is

$$\Pi_V = -c - G + \frac{\binom{M-F-1}{N-1}}{\binom{M-1}{N-1}} [rc - \frac{rc(N-1)Y}{N(M-F-1)}].$$

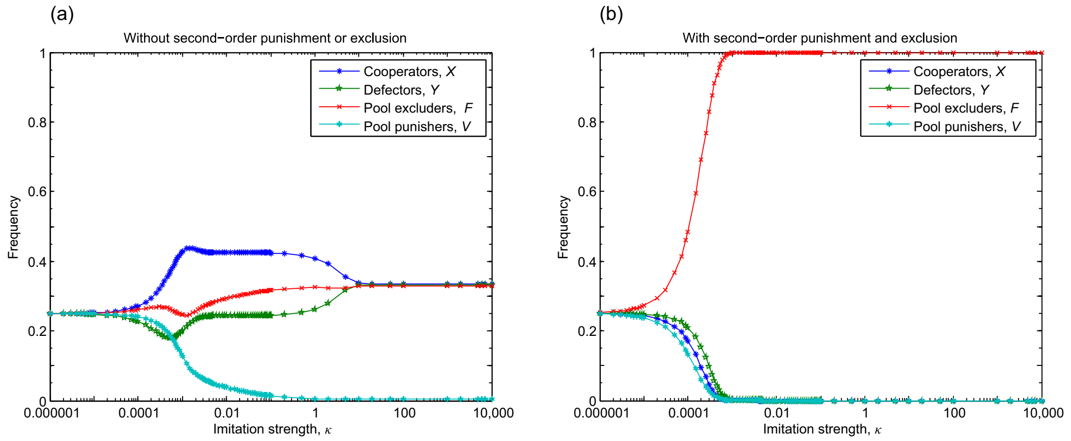

FIG. S1: (Color online) The competition between pool exclusion and pool punishment in the compulsory PGG. In the absence of second-order exclusion and punishment, punishers become extinct, and the other three strategies can coexist in time average (a). In the presence of second-order exclusion and punishment, pool excluders can occupy the whole population (b). Parameters: $N = 5$, $M = 100$, $c = 1$, $r = 3$, $G = 0.4$, $\delta = 0.4$, and $B = 0.4$.

The simplified transition matrix in the strong imitation limit is

$$\begin{pmatrix} \frac{1}{3} & \frac{1}{3} & \frac{1}{3} & 0 \\ 0 & \frac{2}{3} & \frac{1}{3} & 0 \\ 0 & 0 & 1 & 0 \\ 0 & 0 & \frac{1}{3} & \frac{2}{3} \end{pmatrix},$$

which yields the stationary distribution $[0, 0, 1, 0]$. This distribution shows that pool excluders prevail, as shown in Fig. S1(b).

3 Competition between peer exclusion and peer punishment in the optional PGG

In the following let us assume that there are E peer excluders and W peer punishers in the population, and we have $X + Y + Z + E + W = M$. We set that the exclusion probability is 1, then the probability that no peer excluders are found in the population is given by

$$\frac{\binom{M-E-1}{N-1}}{\binom{M-1}{N-1}}.$$

Accordingly, in the absence of second-order exclusion and punishment the average payoff for

cooperators is

$$\Pi_X = \frac{\binom{Z}{N-1}}{\binom{M-1}{N-1}}\sigma + [1 - \frac{\binom{Z}{N-1}}{\binom{M-1}{N-1}}]\{[1 - \frac{\binom{M-E-1}{N-1}}{\binom{M-1}{N-1}}](rc-c) + \frac{\binom{M-E-1}{N-1}}{\binom{M-1}{N-1}} \sum_{i=0}^{N-1} \sum_{k=0}^{N-i-1} \frac{\binom{M-E-Y-Z-1}{i}\binom{Z}{k}\binom{Y}{N-i-k-1}}{\binom{M-E-1}{N-1}}[\frac{rc(i+1)}{N-k} - c]\}$$

The payoff for defectors is

$$\Pi_Y = \frac{\binom{Z}{N-1}}{\binom{M-1}{N-1}}\sigma + [1 - \frac{\binom{Z}{N-1}}{\binom{M-1}{N-1}}]\{[1 - \frac{\binom{M-E-1}{N-1}}{\binom{M-1}{N-1}}][-\frac{(N-1)W\gamma}{M-1}] + \frac{\binom{M-E-1}{N-1}}{\binom{M-1}{N-1}} \sum_{j=0}^{N-1} \sum_{t=0}^{N-1-j} \sum_{k=0}^{N-j-t-1} \frac{\binom{M-E-Y-Z-W}{N-t-k-j-1}\binom{W}{t}\binom{Z}{k}\binom{Y-1}{j}}{\binom{M-E-1}{N-1}}[\frac{rc(N-1-j-k)}{N-k} - t\gamma]\},$$

where t represents the number of peer punishers. The payoff for peer excluders is

$$\Pi_E = \frac{\binom{Z}{N-1}}{\binom{M-1}{N-1}}\sigma + [1 - \frac{\binom{Z}{N-1}}{\binom{M-1}{N-1}}][rc - c - \frac{(N-1)Yc_E}{M-1}].$$

Last, peer punishers' payoff is

$$\Pi_W = \frac{\binom{Z}{N-1}}{\binom{M-1}{N-1}}\sigma + [1 - \frac{\binom{Z}{N-1}}{\binom{M-1}{N-1}}]\{rc - c - \frac{N-1}{M-1}Y\beta - \frac{\binom{M-E-1}{N-1}}{\binom{M-1}{N-1}}[rc - c - \frac{(N-1)Y\beta}{M-1} - \sum_{j=0}^{N-1} \sum_{k=0}^{N-j-1} \frac{\binom{M-E-Y-Z-1}{N-j-k-1}\binom{Z}{k}\binom{Y}{j}}{\binom{M-E-1}{N-1}}(\frac{N-j-k}{N-k}rc - c - j\beta)]\}.$$

The embedded Markov chain describing the transitions between cooperators (X), defectors (Y), loners (Z), peer excluders (E), and peer punishers (W) is given by

$$\begin{pmatrix} I_X & \frac{\rho_{XY}}{4} & \frac{\rho_{XZ}}{4} & \frac{\rho_{XE}}{4} & \frac{\rho_{XW}}{4} \\ \frac{\rho_{YX}}{4} & I_Y & \frac{\rho_{YZ}}{4} & \frac{\rho_{YE}}{4} & \frac{\rho_{YW}}{4} \\ \frac{\rho_{ZX}}{4} & \frac{\rho_{ZY}}{4} & I_Z & \frac{\rho_{ZE}}{4} & \frac{\rho_{ZW}}{4} \\ \frac{\rho_{EX}}{4} & \frac{\rho_{EY}}{4} & \frac{\rho_{EZ}}{4} & I_E & \frac{\rho_{EW}}{4} \\ \frac{\rho_{WX}}{4} & \frac{\rho_{WY}}{4} & \frac{\rho_{WZ}}{4} & \frac{\rho_{WE}}{4} & I_W \end{pmatrix},$$

where $I_K = 1 - \sum_{K \neq L} \frac{\rho_{KL}}{4}$, and $K, L \in \{X, Y, Z, E, W\}$.

For strong imitation, the simplified transitions matrix is

$$\begin{pmatrix} \frac{3}{4} - \frac{1}{2M} & \frac{1}{4} & 0 & \frac{1}{4M} & \frac{1}{4M} \\ 0 & \frac{1}{2} & \frac{1}{4} & \frac{1}{4} & 0 \\ \frac{1}{8} & 0 & \frac{5}{8} & \frac{1}{8} & \frac{1}{8} \\ \frac{1}{4M} & 0 & 0 & 1 - \frac{1}{2M} & \frac{1}{4M} \\ \frac{1}{4M} & 0 & 0 & \frac{1}{4M} & 1 - \frac{1}{2M} \end{pmatrix}.$$

The resulting stationary distribution is $[\frac{6}{5M+23}, \frac{3}{5M+23}, \frac{2}{5M+23}, \frac{3M+6}{5M+23}, \frac{2M+6}{5M+23}]$, which shows that the frequency of peer excluders is higher than the frequency of peer punishers [see Fig.2(a)].

In the presence of second-order exclusion and punishment, we assume that peer excluders exclude peer punishers, pure cooperators, and defectors. Similarly, peer punishers punish peer excluders, pure cooperators, and defectors. The average payoff for cooperators is given by

$$\Pi_X = \frac{\binom{Z}{N-1}}{\binom{M-1}{N-1}}\sigma + [1-\frac{\binom{Z}{N-1}}{\binom{M-1}{N-1}}]\{[1-\frac{\binom{M-E-1}{N-1}}{\binom{M-1}{N-1}}][-c-\frac{(N-1)W\gamma}{M-1}]+ \frac{\binom{M-E-1}{N-1}}{\binom{M-1}{N-1}}\sum_{i=0}^{N-1}\sum_{k=0}^{N-i-1}\sum_{t=0}^{N-i-k-1}\frac{\binom{M-E-W-Y-Z-1}{i}\binom{Z}{k}\binom{W}{t}\binom{Y}{N-i-k-t-1}}{\binom{M-E-1}{N-1}}[\frac{rc(i+t+1)}{N-k}-c-t\gamma]\}.$$

The payoff for defectors is

$$\begin{aligned}\Pi_Y \;=\; & \frac{\binom{Z}{N-1}}{\binom{M-1}{N-1}}\sigma + [1-\frac{\binom{Z}{N-1}}{\binom{M-1}{N-1}}]\{[1-\frac{\binom{M-E-1}{N-1}}{\binom{M-1}{N-1}}][-\frac{(N-1)W\gamma}{M-1}] \\ & +\frac{\binom{M-E-1}{N-1}}{\binom{M-1}{N-1}}\sum_{j=0}^{N-1}\sum_{t=0}^{N-1-j}\sum_{k=0}^{N-j-t-1}\frac{\binom{M-E-Y-Z-W}{N-t-k-j-1}\binom{W}{t}\binom{Z}{k}\binom{Y-1}{j}}{\binom{M-E-1}{N-1}}[\frac{rc(N-1-j-k)}{N-k}-t\gamma]\}.\end{aligned}$$

The payoff for peer excluders is

$$\begin{aligned}\Pi_E \;=\; & \frac{\binom{Z}{N-1}}{\binom{M-1}{N-1}}\sigma + [1-\frac{\binom{Z}{N-1}}{\binom{M-1}{N-1}}] \\ & \sum_{i=0}^{N-1}\sum_{j=0}^{N-1-i}\sum_{k=0}^{N-i-j-1}\sum_{t=0}^{N-i-j-k-1}\frac{\binom{M-X-W-Y-Z-1}{N-i-j-k-t-1}\binom{Z}{k}\binom{X}{i}\binom{W}{t}\binom{Y}{j}}{\binom{M-1}{N-1}}[\frac{r(N-j-k)c}{N-i-j-k-t} \\ & -c-(i+j+t)c_E-t\gamma].\end{aligned}$$

Finally, the payoff for peer punishers is

$$\begin{aligned}\Pi_W \;=\; & \frac{\binom{Z}{N-1}}{\binom{M-1}{N-1}}\sigma + [1-\frac{\binom{Z}{N-1}}{\binom{M-1}{N-1}}]\{[1-\frac{\binom{M-E-1}{N-1}}{\binom{M-1}{N-1}}][-c-\frac{(N-1)(X+E+Y)\beta}{M-1}]+ \\ & \frac{\binom{M-E-1}{N-1}}{\binom{M-1}{N-1}}\sum_{i=0}^{N-1}\sum_{j=0}^{N-1-i}\sum_{k=0}^{N-i-j-1}\frac{\binom{M-E-X-Y-Z-1}{N-i-j-k-1}\binom{Z}{k}\binom{X}{i}\binom{Y}{j}}{\binom{M-E-1}{N-1}}[\frac{rc(N-j-k)}{N-k}-c-(i+j)\beta]\}.\end{aligned}$$

The transition matrix between cooperators, defectors, loners, peer excluders, and peer punishers in the strong imitation limit is

$$\begin{pmatrix} \frac{1}{2} & \frac{1}{4} & 0 & \frac{1}{4} & 0 \\ 0 & \frac{1}{2} & \frac{1}{4} & \frac{1}{4} & 0 \\ \frac{1}{8} & 0 & \frac{5}{8} & \frac{1}{8} & \frac{1}{8} \\ 0 & 0 & 0 & 1 & 0 \\ 0 & 0 & 0 & \frac{1}{4} & \frac{3}{4} \end{pmatrix},$$

which yields the $[0,0,0,1,0]$ stationary distribution. As a result, peer excluders prevail, shown in Fig.2(b).

4 Competition between peer exclusion and peer punishment in the compulsory PGG

Considering the compulsory PGG the probability that no peer excluder is found in the group is

$$\frac{\binom{M-E-1}{N-1}}{\binom{M-1}{N-1}}.$$

In the absence of second-order exclusion and punishment, the average payoff for cooperators is

$$\begin{aligned}\Pi_X &= [1-\frac{\binom{M-E-1}{N-1}}{\binom{M-1}{N-1}}](rc-c)+\frac{\binom{M-E-1}{N-1}}{\binom{M-1}{N-1}}\sum_{i=0}^{N-1}\frac{\binom{M-E-Y-1}{i}\binom{Y}{N-i-1}}{\binom{M-E-1}{N-1}}[\frac{rc(i+1)}{N}-c]\\ &= rc[1-\frac{\binom{M-E-1}{N-1}}{\binom{M-1}{N-1}}\frac{(N-1)Y}{(M-E-1)N}]-c,\end{aligned}$$

while the average payoff for defectors is

$$\Pi_Y=\frac{\binom{M-E-1}{N-1}}{\binom{M-1}{N-1}}\frac{rc(M-1)(N-1)(W+X)-N(N-1)WE\gamma}{N(M-E-1)(M-1)}-\frac{(N-1)W\gamma}{M-1}.$$

The average payoff for peer excluders is

$$\begin{aligned}\Pi_E &= \sum_{i=0}^{N-1}\frac{\binom{M-Y-1}{i}\binom{Y}{N-i-1}}{\binom{M-1}{N-1}}[rc-c-(N-i-1)c_E]\\ &= rc-c-\frac{(N-1)Yc_E}{M-1}.\end{aligned}$$

Last, the payoff for peer punishers is

$$\Pi_W=rc-c-\frac{(N-1)Y\beta}{M-1}-\frac{\binom{M-E-1}{N-1}}{\binom{M-1}{N-1}}\frac{rc(M-1)(N-1)Y+(N-1)NEY\beta}{N(M-E-1)(M-1)}.$$

For small mutation rate, the embedded Markov chain describing the transitions among cooperators (X), defectors (Y), peer excluders (E), and peer punishers (W) is given by

$$\begin{pmatrix} I_X & \frac{\rho_{XY}}{3} & \frac{\rho_{XE}}{3} & \frac{\rho_{XW}}{3}\\ \frac{\rho_{YX}}{3} & I_Y & \frac{\rho_{YE}}{3} & \frac{\rho_{YW}}{3}\\ \frac{\rho_{EX}}{3} & \frac{\rho_{EY}}{3} & I_E & \frac{\rho_{EW}}{3}\\ \frac{\rho_{WX}}{3} & \frac{\rho_{WY}}{3} & \frac{\rho_{WE}}{3} & I_W \end{pmatrix},$$

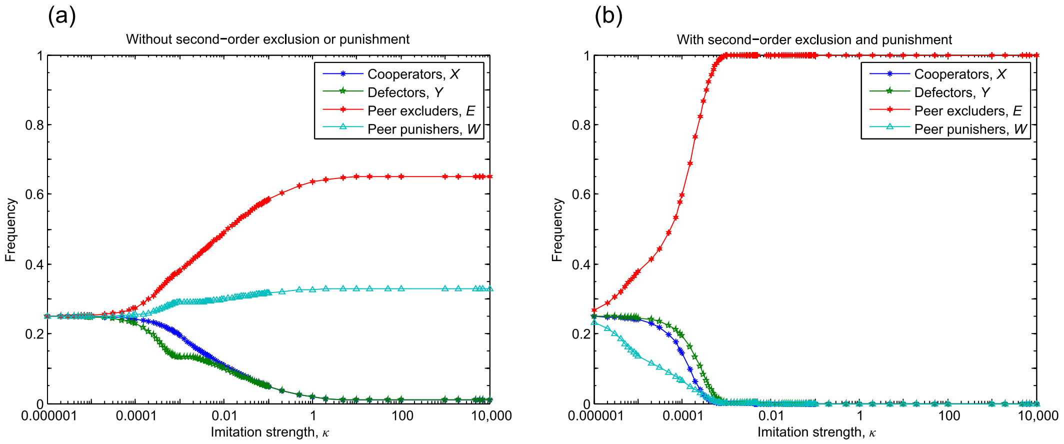

FIG. S2: (Color online) The competition between peer exclusion and peer punishment in the compulsory PGG. In the absence of second-order exclusion and punishment (a), the final frequency of peer excluders is about two times higher than that of peer punishers, and other two strategists become extinct (a). With second-order exclusion and punishment, peer excluders win (b). Parameters: $N = 5$, $M = 100$, $c = 1$, $r = 3$, $c_E = 0.4$, $\beta = 0.4$, and $\gamma = 0.4$.

where $I_K = 1 - \sum_{K \neq L} \frac{\rho_{KL}}{3}$, and $K, L \in \{X, Y, E, W\}$.

The simplified transition matrix between cooperators (X), defectors (Y), peer excluders (E), and peer punishers (W) in the strong imitation can be given as

$$\begin{pmatrix} \frac{2}{3} - \frac{2}{3M} & \frac{1}{3} & \frac{1}{3M} & \frac{1}{3M} \\ 0 & \frac{2}{3} & \frac{1}{3} & 0 \\ \frac{1}{3M} & 0 & 1 - \frac{2}{3M} & \frac{1}{3M} \\ \frac{1}{3M} & 0 & \frac{1}{3M} & 1 - \frac{2}{3M} \end{pmatrix}.$$

The resulting stationary distribution is $[\frac{1}{4+M}, \frac{1}{4+M}, \frac{2M+3}{12+3M}, \frac{M+3}{12+3M}]$, which shows that peer excluders' frequency is higher than that of other strategists (see Fig.S2).

If we consider second-order exclusion and punishment, we assume that peer excluders exclude peer punishers, pure cooperators, and defectors. Simultaneously, peer punishers punish peer excluders, pure cooperators, and defectors. Accordingly, the payoff for defectors remains unchanged, but the average payoff for cooperators is changed to

$$\Pi_X = -c - \frac{(N-1)W\gamma}{M-1} + \frac{\binom{M-E-1}{N-1}}{\binom{M-1}{N-1}} \{ \frac{rc(M-1)[N(M-E-1)-(N-1)Y] - NE(N-1)W\gamma}{N(M-1)(M-E-1)} \}.$$

The payoff for peer excluders is

$$\Pi_E = \sum_{i=0}^{N-1}\sum_{j=0}^{N-1-i}\sum_{t=0}^{N-i-j-1} \frac{\binom{M-W-Y-X-1}{N-i-j-t-1}\binom{X}{i}\binom{W}{t}\binom{Y}{j}}{\binom{M-1}{N-1}}[\frac{r(N-j)c}{N-i-j-t} - c - (i+j+t)c_E - t\gamma],$$

while the payoff for peer punishers is

$$\begin{aligned}\Pi_W &= -c - \frac{(N-1)(X+E+Y)\beta}{M-1} + \frac{\binom{M-E-1}{N-1}}{\binom{M-1}{N-1}}[rc - \frac{(N-1)Yrc}{N(M-E-1)} - \\ &\frac{(N-1)(M-W-E)\beta}{M-E-1} + \frac{(N-1)(X+E+Y)\beta}{M-1}].\end{aligned}$$

Here the transitions matrix between cooperators, defectors, peer excluders, and peer punishers is

$$\begin{pmatrix} \frac{1}{3} & \frac{1}{3} & \frac{1}{3} & 0 \\ 0 & \frac{2}{3} & \frac{1}{3} & 0 \\ 0 & 0 & 1 & 0 \\ 0 & 0 & \frac{1}{3} & \frac{2}{3} \end{pmatrix},$$

which gives the stationary distribution $(0, 0, 1, 0)$. It suggests that peer excluders prevail as shown in Fig. S2.

5 Competition between pool exclusion and peer exclusion in the optional PGG

In the optional PGG, the probability that no peer excluder or pool excluder is found in the group is given by

$$\frac{\binom{M-E-F-1}{N-1}}{\binom{M-1}{N-1}}.$$

In the absence of second-order exclusion, the average payoff for cooperators is given by

$$\begin{aligned}\Pi_X &= \frac{\binom{Z}{N-1}}{\binom{M-1}{N-1}}\sigma + [1 - \frac{\binom{Z}{N-1}}{\binom{M-1}{N-1}}]\{[1 - \frac{\binom{M-E-F-1}{N-1}}{\binom{M-1}{N-1}}](rc-c) + \\ &\frac{\binom{M-E-F-1}{N-1}}{\binom{M-1}{N-1}}\sum_{i=0}^{N-1}\sum_{k=0}^{N-1-i}\frac{\binom{M-E-F-Y-Z-1}{i}\binom{Z}{k}\binom{Y}{N-i-k-1}}{\binom{M-E-F-1}{N-1}}[\frac{rc(i+1)}{N-k} - c]\}.\end{aligned}$$

The average payoff for defectors is

$$\Pi_Y = \frac{\binom{Z}{N-1}}{\binom{M-1}{N-1}}\sigma + [1 - \frac{\binom{Z}{N-1}}{\binom{M-1}{N-1}}]\frac{\binom{M-E-F-1}{N-1}}{\binom{M-1}{N-1}}\sum_{i=0}^{N-1}\sum_{k=0}^{N-1-i}\frac{\binom{M-E-F-Y-Z}{i}\binom{Z}{k}\binom{Y-1}{N-i-k-1}}{\binom{M-F-E-1}{N-1}}\frac{ric}{N-k}.$$

The average payoff for pool excluders is

$$\Pi_F = \frac{\binom{Z}{N-1}}{\binom{M-1}{N-1}}\sigma + [1 - \frac{\binom{Z}{N-1}}{\binom{M-1}{N-1}}](rc - c - \delta).$$

The average payoff for peer excluders is

$$\Pi_E = \frac{\binom{Z}{N-1}}{\binom{M-1}{N-1}}\sigma + [1 - \frac{\binom{Z}{N-1}}{\binom{M-1}{N-1}}][rc - c - \frac{(N-1)Yc_E}{M-1}].$$

For small mutation rate, the embedded Markov chain describing the transitions between cooperators (X), defectors (Y), loners (Z), pool excluders (F), and peer excluders (E) is given by

$$\begin{pmatrix} I_X & \frac{\rho_{XY}}{4} & \frac{\rho_{XZ}}{4} & \frac{\rho_{XF}}{4} & \frac{\rho_{XE}}{4} \\ \frac{\rho_{YX}}{4} & I_Y & \frac{\rho_{YZ}}{4} & \frac{\rho_{YF}}{4} & \frac{\rho_{YE}}{4} \\ \frac{\rho_{ZX}}{4} & \frac{\rho_{ZY}}{4} & I_Z & \frac{\rho_{ZF}}{4} & \frac{\rho_{ZE}}{4} \\ \frac{\rho_{FX}}{4} & \frac{\rho_{FY}}{4} & \frac{\rho_{FZ}}{4} & I_F & \frac{\rho_{FE}}{4} \\ \frac{\rho_{EX}}{4} & \frac{\rho_{EY}}{4} & \frac{\rho_{EZ}}{4} & \frac{\rho_{EF}}{4} & I_E \end{pmatrix},$$

where $I_K = 1 - \sum_{K \neq L} \frac{\rho_{KL}}{4}$, and $K, L \in \{X, Y, Z, F, E\}$.

For strong imitation, the transition matrix becomes to

$$\begin{pmatrix} \frac{3}{4} - \frac{1}{4M} & \frac{1}{4} & 0 & 0 & \frac{1}{4M} \\ 0 & \frac{1}{4} & \frac{1}{4} & \frac{1}{4} & \frac{1}{4} \\ \frac{1}{8} & 0 & \frac{5}{8} & \frac{1}{8} & \frac{1}{8} \\ \frac{1}{4} & 0 & 0 & \frac{1}{2} & \frac{1}{4} \\ \frac{1}{4M} & 0 & 0 & 0 & 1 - \frac{1}{4M} \end{pmatrix}.$$

Accordingly, the stationary distribution is $[\frac{9}{25+6M}, \frac{3}{25+6M}, \frac{2}{25+6M}, \frac{2}{25+6M}, \frac{6M+9}{25+6M}]$, and the population is dominated by peer excluders (see Fig. 3).

If we consider second-order exclusion, we assume that pool excluders exclude peer excluders because the latter players do not contribute to the exclusion pool. But peer excluders do not exclude pool excluders. Accordingly, the average payoff for cooperators is given by

$$\begin{aligned} \Pi_X \ = \ & \frac{\binom{Z}{N-1}}{\binom{M-1}{N-1}}\sigma + [1 - \frac{\binom{Z}{N-1}}{\binom{M-1}{N-1}}]\{[1 - \frac{\binom{M-E-F-1}{N-1}}{\binom{M-1}{N-1}}](-c) + \\ & \frac{\binom{M-E-F-1}{N-1}}{\binom{M-1}{N-1}} \sum_{i=0}^{N-1} \sum_{k=0}^{N-1-i} \frac{\binom{M-E-F-Y-Z-1}{i}\binom{Z}{k}\binom{Y}{N-i-k-1}}{\binom{M-E-F-1}{N-1}}[\frac{rc(i+1)}{N-k} - c]\}, \end{aligned}$$

where $\frac{\binom{M-E-F-1}{N-1}}{\binom{M-1}{N-1}}$ denotes the probability that neither peer excluder nor pool excluder is found in the group. The average payoffs for peer excluders and pool excluders are respectively

$$\begin{aligned}\Pi_E \;=\; &\frac{\binom{Z}{N-1}}{\binom{M-1}{N-1}}\sigma + [1-\frac{\binom{Z}{N-1}}{\binom{M-1}{N-1}}]\{[1-\frac{\binom{M-F-1}{N-1}}{\binom{M-1}{N-1}}][-c-\sum_{i=0}^{N-1}\sum_{j=0}^{N-1-i}\frac{\binom{M-Y-X-1}{N-i-j-1}\binom{X}{i}\binom{Y}{j}}{\binom{M-1}{N-1}}(i+j)c_E]\\ &+\frac{\binom{M-F-1}{N-1}}{\binom{M-1}{N-1}}\sum_{i=0}^{N-1}\sum_{k=0}^{N-1-i}\sum_{j=0}^{N-1-i-k}\frac{\binom{M-X-F-Y-Z-1}{N-i-j-k-1}\binom{Z}{k}\binom{Y}{j}\binom{X}{i}}{\binom{M-F-1}{N-1}}[\frac{rc(N-j-k)}{N-j-i-k}-c-(i+j)c_E]\},\end{aligned}$$

$$\Pi_F = \frac{\binom{Z}{N-1}}{\binom{M-1}{N-1}}\sigma + [1-\frac{\binom{Z}{N-1}}{\binom{M-1}{N-1}}]\sum_{k=0}^{N-1}\sum_{j=0}^{N-1-k}\sum_{l=0}^{N-1-k-j}\frac{\binom{M-F-Y-Z}{N-j-k-l-1}\binom{Z}{k}\binom{Y}{j}\binom{F-1}{l}}{\binom{M-1}{N-1}}[\frac{rc(N-j-k)}{l+1}-c-\delta].$$

The payoffs of loners and defectors are not changed.

For strong imitation, the transitions between cooperators (X), defectors (Y), loners (Z), pool excluders (F), and peer excluders (E) are given by

$$\begin{pmatrix} \frac{1}{4} & \frac{1}{4} & 0 & \frac{1}{4} & \frac{1}{4} \\ 0 & \frac{1}{4} & \frac{1}{4} & \frac{1}{4} & \frac{1}{4} \\ \frac{1}{8} & 0 & \frac{5}{8} & \frac{1}{8} & \frac{1}{8} \\ 0 & 0 & 0 & 1 & 0 \\ 0 & 0 & 0 & \frac{1}{4} & \frac{3}{4} \end{pmatrix}.$$

Accordingly, the stationary distribution is $[0, 0, 0, 1, 0]$, which shows that pool excluders prevail (see Fig. 3).

6 Competition between pool exclusion and peer exclusion in the compulsory PGG

In the compulsory PGG, the probability that no peer excluder or no pool excluder is found in the group is given by

$$\frac{\binom{M-E-F-1}{N-1}}{\binom{M-1}{N-1}}.$$

In the absence of second-order exclusion, the average payoff for cooperators is given by

$$\begin{aligned}\Pi_X &= [1-\frac{\binom{M-E-F-1}{N-1}}{\binom{M-1}{N-1}}](rc-c) + \frac{\binom{M-E-F-1}{N-1}}{\binom{M-1}{N-1}}\sum_{i=0}^{N-1}\frac{\binom{M-E-F-Y-1}{i}\binom{Y}{N-i-1}}{\binom{M-E-F-1}{N-1}}[\frac{rc(i+1)}{N}-c]\\ &= rc[1-\frac{\binom{M-E-F-1}{N-1}}{\binom{M-1}{N-1}}\frac{(N-1)Y}{N(M-E-F-1)}]-c.\end{aligned}$$

The payoff for defectors is

$$\Pi_Y = \frac{\binom{M-E-F-1}{N-1}}{\binom{M-1}{N-1}} \sum_{i=0}^{N-1} \frac{\binom{M-E-F-Y}{i}\binom{Y-1}{N-i-1}}{\binom{M-E-F-1}{N-1}} \frac{rci}{N}$$
$$= \frac{\binom{M-E-F-1}{N-1}}{\binom{M-1}{N-1}} \frac{rc}{N} \frac{(N-1)(M-E-F-Y)}{M-E-F-1},$$

while the average payoff for pool excluders is

$$\Pi_F = rc - c - \delta.$$

Last, the average payoff for peer excluders is

$$\Pi_E = rc - c - \frac{(N-1)Yc_E}{M-1}.$$

For small mutation rate, the embedded Markov chain describing the transitions between cooperators (X), defectors (Y), pool excluders (F), and peer excluders (E) is given by

$$\begin{pmatrix} I_X & \frac{\rho_{XY}}{3} & \frac{\rho_{XF}}{3} & \frac{\rho_{XE}}{3} \\ \frac{\rho_{YX}}{3} & I_Y & \frac{\rho_{YF}}{3} & \frac{\rho_{YE}}{3} \\ \frac{\rho_{FX}}{3} & \frac{\rho_{FY}}{3} & I_F & \frac{\rho_{FE}}{3} \\ \frac{\rho_{EX}}{3} & \frac{\rho_{EY}}{3} & \frac{\rho_{EF}}{3} & I_E \end{pmatrix},$$

where $I_K = 1 - \sum_{K \neq L} \frac{\rho_{KL}}{3}$, and $K, L \in \{X, Y, F, E\}$.

For strong imitation, the transition matrix becomes

$$\begin{pmatrix} \frac{2}{3} - \frac{1}{3M} & \frac{1}{3} & 0 & \frac{1}{3M} \\ 0 & \frac{1}{3} & \frac{1}{3} & \frac{1}{3} \\ \frac{1}{3} & 0 & \frac{1}{3} & \frac{1}{3} \\ \frac{1}{3M} & 0 & 0 & 1 - \frac{1}{3M} \end{pmatrix}.$$

The resulting stationary distribution is $[\frac{4}{3M+11}, \frac{2}{3M+11}, \frac{1}{3M+11}, \frac{3M+4}{3M+11}]$, which suggests that the population is dominated by peer excluders (see Fig.S3).

In the presence of second-order exclusion, we assume that pool excluders exclude peer excluders, but peer excluders do not exclude pool excluders. The average payoff for cooperators is given by

$$\Pi_X = [1 - \frac{\binom{M-E-F-1}{N-1}}{\binom{M-1}{N-1}}](-c) + \frac{\binom{M-E-F-1}{N-1}}{\binom{M-1}{N-1}} \sum_{i=0}^{N-1} \frac{\binom{M-E-F-Y-1}{i}\binom{Y}{N-i-1}}{\binom{M-E-F-1}{N-1}} [\frac{rc(i+1)}{N} - c]$$
$$= \frac{\binom{M-E-F-1}{N-1}}{\binom{M-1}{N-1}} \{\frac{rc}{N}[\frac{(M-Y-E-F-1)(N-1)}{M-E-F-1} + 1]\} - c.$$

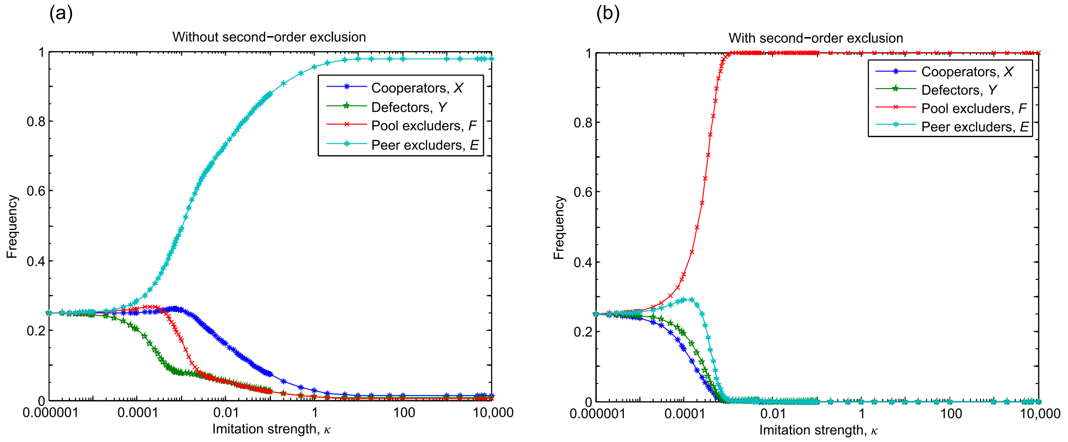

FIG. S3: (Color online) The competition between peer and pool exclusion in the compulsory PGG. In the absence of second-order exclusion, peer excluders can occupy the whole population, and other strategists become extinct (a). With second-order exclusion, pool excluders win (b). Parameters: $N = 5$, $M = 100$, $c = 1$, $r = 3$, $c_E = 0.4$, and $\delta = 0.4$.

The average payoffs for pool excluders and peer excluders are respectively given by

$$\Pi_F = \sum_{l=0}^{N-1}\sum_{i=0}^{N-l-1} \frac{\binom{M-X-E-Y-1}{l}\binom{Y}{N-i-l-1}\binom{X+E}{i}}{\binom{M-1}{N-1}}[\frac{rc(i+l+1)}{l+1} - c - \delta],$$

$$\Pi_E = [1 - \frac{\binom{M-F-1}{N-1}}{\binom{M-1}{N-1}}][-c - \frac{(N-1)(X+Y)}{M-1}c_E] + \frac{\binom{M-F-1}{N-1}}{\binom{M-1}{N-1}} \sum_{i=0}^{N-1}\sum_{j=0}^{N-i-1} \frac{\binom{M-X-F-Y-1}{N-j-i-1}\binom{Y}{j}\binom{X}{i}}{\binom{M-F-1}{N-1}}[\frac{rc(N-j)}{N-i-j} - c - (i+j)c_E].$$

For strong imitation, the transition matrix is

$$\begin{pmatrix} 0 & \frac{1}{3} & \frac{1}{3} & \frac{1}{3} \\ 0 & \frac{1}{3} & \frac{1}{3} & \frac{1}{3} \\ 0 & 0 & 1 & 0 \\ 0 & 0 & \frac{1}{3} & \frac{2}{3} \end{pmatrix}.$$

The resulting stationary distribution is $[0, 0, 1, 0]$, namely pool excluders prevail (see Fig. S3).

7 Competition between prosocial exclusions and punishments in the optional PGG

In this case the population contains X cooperators, Y defectors, Z loners, E peer excluders, W peer punishers, F pool excluders, and V pool punishers whose fractions fulfill the constraint

$X + Y + Z + E + W + F + V = M$. In the absence of second-order exclusion and punishment, the average payoff for cooperators is

$$\begin{aligned}\Pi_X \;=\; & \frac{\binom{Z}{N-1}}{\binom{M-1}{N-1}}\sigma + [1 - \frac{\binom{Z}{N-1}}{\binom{M-1}{N-1}}]\{[1 - \frac{\binom{M-E-F-1}{N-1}}{\binom{M-1}{N-1}}](rc - c) + \\ & \frac{\binom{M-E-F-1}{N-1}}{\binom{M-1}{N-1}} \sum_{i=0}^{N-1} \sum_{k=0}^{N-1-i} \frac{\binom{M-E-F-Y-Z-1}{i}\binom{Z}{k}\binom{Y}{N-i-k-1}}{\binom{M-E-F-1}{N-1}}[\frac{rc(i+1)}{N-k} - c]\}.\end{aligned}$$

The payoff for defectors is

$$\begin{aligned}\Pi_Y = & \frac{\binom{Z}{N-1}}{\binom{M-1}{N-1}}\sigma + [1 - \frac{\binom{Z}{N-1}}{\binom{M-1}{N-1}}]\{[1 - \frac{\binom{M-E-F-1}{N-1}}{\binom{M-1}{N-1}}][-\frac{(N-1)W\gamma + (N-1)VB}{M-1}] + \frac{\binom{M-E-F-1}{N-1}}{\binom{M-1}{N-1}} \\ & \sum_{j=0}^{N-1} \sum_{t=0}^{N-1-j} \sum_{k=0}^{N-1-j-t} \sum_{p=0}^{N-1-j-t-k} \frac{\binom{M-E-F-Y-Z-W-V}{N-j-t-k-p-1}\binom{W}{t}\binom{Z}{k}\binom{V}{p}\binom{Y-1}{j}}{\binom{M-F-E-1}{N-1}}[\frac{r(N-j-k-1)c}{N-k} - t\gamma - pB]\}.\end{aligned}$$

The average payoff for peer excluders is

$$\Pi_E = \frac{\binom{Z}{N-1}}{\binom{M-1}{N-1}}\sigma + [1 - \frac{\binom{Z}{N-1}}{\binom{M-1}{N-1}}][rc - c - \frac{(N-1)Yc_E}{M-1}].$$

The average payoff for peer punishers is

$$\begin{aligned}\Pi_W \;=\; & \frac{\binom{Z}{N-1}}{\binom{M-1}{N-1}}\sigma + [1 - \frac{\binom{Z}{N-1}}{\binom{M-1}{N-1}}]\{[1 - \frac{\binom{M-E-F-1}{N-1}}{\binom{M-1}{N-1}}][rc - c - \frac{(N-1)Y\beta}{M-1}] \\ & + \frac{\binom{M-E-F-1}{N-1}}{\binom{M-1}{N-1}} \sum_{i=0}^{N-1} \sum_{k=0}^{N-1-i} \frac{\binom{M-E-F-Y-Z-1}{i}\binom{Z}{k}\binom{Y}{N-i-k-1}}{\binom{M-F-E-1}{N-1}}[\frac{rc(i+1)}{N-k} - c - (N-i-k-1)\beta]\}.\end{aligned}$$

The average payoff for pool excluders is

$$\Pi_F = \frac{\binom{Z}{N-1}}{\binom{M-1}{N-1}}\sigma + [1 - \frac{\binom{Z}{N-1}}{\binom{M-1}{N-1}}](rc - c - \delta).$$

Last, the average payoff for pool punishers is

$$\begin{aligned}\Pi_V \;=\; & \frac{\binom{Z}{N-1}}{\binom{M-1}{N-1}}\sigma + [1 - \frac{\binom{Z}{N-1}}{\binom{M-1}{N-1}}]\{[1 - \frac{\binom{M-E-F-1}{N-1}}{\binom{M-1}{N-1}}](rc - c - G) \\ & + \frac{\binom{M-E-F-1}{N-1}}{\binom{M-1}{N-1}} \sum_{j=0}^{N-1} \sum_{k=0}^{N-j-1} \frac{\binom{M-E-F-Y-Z-1}{N-j-k-1}\binom{Y}{j}\binom{Z}{k}}{\binom{M-E-F-1}{N-1}}[\frac{r(N-j-k)c}{N-k} - c - G]\}.\end{aligned}$$

For small exploration rates, the embedded Markov chain describing the transitions between cooperators (X), defectors (Y), loners (Z), peer excluders (E), peer punishers (W), pool excluders

(F), and pool punishers (V) is given by

$$\begin{pmatrix} I_X & \frac{\rho_{XY}}{6} & \frac{\rho_{XZ}}{6} & \frac{\rho_{XE}}{6} & \frac{\rho_{XW}}{6} & \frac{\rho_{XF}}{6} & \frac{\rho_{XV}}{6} \\ \frac{\rho_{YX}}{6} & I_Y & \frac{\rho_{YZ}}{6} & \frac{\rho_{YE}}{6} & \frac{\rho_{YW}}{6} & \frac{\rho_{YF}}{6} & \frac{\rho_{YV}}{6} \\ \frac{\rho_{ZX}}{6} & \frac{\rho_{ZY}}{6} & I_Z & \frac{\rho_{ZE}}{6} & \frac{\rho_{ZW}}{6} & \frac{\rho_{ZF}}{6} & \frac{\rho_{ZV}}{6} \\ \frac{\rho_{EX}}{6} & \frac{\rho_{EY}}{6} & \frac{\rho_{EZ}}{6} & I_E & \frac{\rho_{EW}}{6} & \frac{\rho_{EF}}{6} & \frac{\rho_{EV}}{6} \\ \frac{\rho_{WX}}{6} & \frac{\rho_{WY}}{6} & \frac{\rho_{WZ}}{6} & \frac{\rho_{WE}}{6} & I_W & \frac{\rho_{WF}}{6} & \frac{\rho_{WV}}{6} \\ \frac{\rho_{FX}}{6} & \frac{\rho_{FY}}{6} & \frac{\rho_{FZ}}{6} & \frac{\rho_{FE}}{6} & \frac{\rho_{FW}}{6} & I_F & \frac{\rho_{FV}}{6} \\ \frac{\rho_{VX}}{6} & \frac{\rho_{VY}}{6} & \frac{\rho_{VZ}}{6} & \frac{\rho_{VE}}{6} & \frac{\rho_{VW}}{6} & \frac{\rho_{VF}}{6} & I_V \end{pmatrix},$$

where $I_K = 1 - \sum_{K \neq L} \frac{\rho_{KL}}{6}$, and $K, L \in \{X, Y, Z, E, W, F, V\}$.

For strong imitation, the transitions matrix is

$$\begin{pmatrix} \frac{5}{6} - \frac{1}{3M} & \frac{1}{6} & 0 & \frac{1}{6M} & \frac{1}{6M} & 0 & 0 \\ 0 & \frac{1}{2} & \frac{1}{6} & \frac{1}{6} & 0 & \frac{1}{6} & 0 \\ \frac{1}{12} & 0 & \frac{7}{12} & \frac{1}{12} & \frac{1}{12} & \frac{1}{12} & \frac{1}{12} \\ \frac{1}{6M} & 0 & 0 & 1 - \frac{1}{3M} & \frac{1}{6M} & 0 & 0 \\ \frac{1}{6M} & 0 & 0 & \frac{1}{6M} & 1 - \frac{1}{3M} & 0 & 0 \\ \frac{1}{6} & 0 & 0 & \frac{1}{6} & \frac{1}{6} & \frac{1}{2} - \frac{1}{6M} & \frac{1}{6M} \\ \frac{1}{6} & 0 & 0 & \frac{1}{6} & \frac{1}{6} & \frac{1}{6M} & \frac{1}{2} - \frac{1}{6M} \end{pmatrix}.$$

Accordingly, the stationary distribution is $[\frac{45}{163+35M}, \frac{15}{163+35M}, \frac{6}{163+35M}, \frac{20M+45}{163+35M}, \frac{15M+45}{163+35M}, \frac{18M+7}{(163+35M)(3M+2)}, \frac{3M+7}{(163+35M)(3M+2)}]$, suggesting that the frequency of peer excluders is higher than the fraction of any other strategy (see Fig.4).

If we consider the second-order exclusion and punishment, then the average payoff for cooperators is given by

$$\Pi_X = \frac{\binom{Z}{N-1}}{\binom{M-1}{N-1}}\sigma + [1 - \frac{\binom{Z}{N-1}}{\binom{M-1}{N-1}}]\{[1 - \frac{\binom{M-E-F-1}{N-1}}{\binom{M-1}{N-1}}][-c - \frac{(N-1)W\gamma}{M-1} - \frac{(N-1)VB}{M-1}] +$$
$$\frac{\binom{M-E-F-1}{N-1}}{\binom{M-1}{N-1}} \sum_{i=0}^{N-1} \sum_{t=0}^{N-1-i} \sum_{p=0}^{N-1-i-t} \sum_{k=0}^{N-1-i-t-p} \frac{\binom{M-E-F-V-Y-W-Z-1}{i}\binom{Y}{N-i-t-p-k-1}\binom{W}{t}\binom{V}{p}\binom{Z}{k}}{\binom{M-E-F-1}{N-1}}$$
$$\times [\frac{rc(i+t+p+1)}{N-k} - c - t\gamma - pB]\}.$$

The payoff for peer excluders is

$$\Pi_E = \frac{\binom{Z}{N-1}}{\binom{M-1}{N-1}}\sigma + [1 - \frac{\binom{Z}{N-1}}{\binom{M-1}{N-1}}]\{[1 - \frac{\binom{M-F-1}{N-1}}{\binom{M-1}{N-1}}][-c-$$
$$\frac{W\gamma + VB + (X+Y+W+V)c_E}{M-1}(N-1)] + \frac{\binom{M-F-1}{N-1}}{\binom{M-1}{N-1}}$$
$$\sum_{i=0}^{N-1}\sum_{j=0}^{N-1-i}\sum_{k=0}^{N-1-i-j}\sum_{p=0}^{N-1-i-j-k}\sum_{t=0}^{N-1-i-j-k-p}\frac{\binom{M-F-X-W-Y-Z-V-1}{N-i-j-k-p-t-1}\binom{X}{i}\binom{Y}{j}\binom{Z}{k}\binom{W}{t}\binom{V}{p}}{\binom{M-F-1}{N-1}}$$
$$\times[\frac{rc(N-j-k)}{N-i-j-k-p-t} - t\gamma - pB - (i+j+t+p)c_E - c]\}.$$

The average payoff for peer punishers is

$$\Pi_W = \frac{\binom{Z}{N-1}}{\binom{M-1}{N-1}}\sigma + [1 - \frac{\binom{Z}{N-1}}{\binom{M-1}{N-1}}]\{[1 - \frac{\binom{M-E-F-1}{N-1}}{\binom{M-1}{N-1}}][-c-$$
$$(N-1)\frac{VB + (X+Y+E+F)\beta}{M-1}] + \frac{\binom{M-E-F-1}{N-1}}{\binom{M-1}{N-1}}$$
$$\sum_{i=0}^{N-1}\sum_{j=0}^{N-i-1}\sum_{k=0}^{N-i-j-1}\sum_{p=0}^{N-i-j-k-1}\frac{\binom{M-F-X-Z-V-Y-E-1}{N-i-j-p-k-1}\binom{V}{p}\binom{X}{i}\binom{Z}{k}\binom{Y}{j}}{\binom{M-E-F-1}{N-1}}$$
$$\times[\frac{rc(N-j-k)}{N-k} - c - (i+j)\beta - pB]\}.$$

The average payoff for pool excluders is

$$\Pi_F = \frac{\binom{Z}{N-1}}{\binom{M-1}{N-1}}\sigma + [1 - \frac{\binom{Z}{N-1}}{\binom{M-1}{N-1}}]$$
$$\sum_{j=0}^{N-1}\sum_{t=0}^{N-j-1}\sum_{p=0}^{N-t-j-1}\sum_{l=0}^{N-t-j-p-1}\frac{\binom{M-F-Z-V-Y-W}{N-t-j-p-l-1}\binom{V}{p}\binom{Y+Z}{j}\binom{W}{t}\binom{F-1}{l}}{\binom{M-1}{N-1}}$$
$$\times[\frac{rc(N-j)}{l+1} - c - t\gamma - pB - \delta]\}.$$

Last, the payoff for pool punishers is

$$\Pi_V = \frac{\binom{Z}{N-1}}{\binom{M-1}{N-1}}\sigma + [1 - \frac{\binom{Z}{N-1}}{\binom{M-1}{N-1}}]\{[1 - \frac{\binom{M-E-F-1}{N-1}}{\binom{M-1}{N-1}}](-c-G) + \frac{\binom{M-E-F-1}{N-1}}{\binom{M-1}{N-1}}$$
$$\sum_{j=0}^{N-1}\sum_{k=0}^{N-j-1}\frac{\binom{Y}{j}\binom{Z}{k}\binom{M-E-F-Y-Z-1}{N-j-k-1}}{\binom{M-E-F-1}{N-1}}[\frac{r(N-j-k)c}{N-k} - c - G]\}.$$

For strong imitation limit, the embedded Markov chain describing the transition matrix between cooperators (X), defectors (Y), loners (Z), peer excluders (E), peer punishers (W), pool excluders

(F), and pool punishers (V) is

$$\begin{pmatrix} \frac{1}{2} & \frac{1}{6} & 0 & \frac{1}{6} & 0 & \frac{1}{6} & 0 \\ 0 & \frac{1}{2} & \frac{1}{6} & \frac{1}{6} & 0 & \frac{1}{6} & 0 \\ \frac{1}{12} & 0 & \frac{7}{12} & \frac{1}{12} & \frac{1}{12} & \frac{1}{12} & \frac{1}{12} \\ 0 & 0 & 0 & \frac{5}{6} & 0 & \frac{1}{6} & 0 \\ 0 & 0 & 0 & \frac{1}{6} & \frac{2}{3} & \frac{1}{6} & 0 \\ 0 & 0 & 0 & 0 & 0 & 1 & 0 \\ 0 & 0 & 0 & \frac{1}{6} & 0 & \frac{1}{6} & \frac{2}{3} \end{pmatrix}. \quad (1)$$

The resulting stationary distribution $[0, 0, 0, 0, 0, 1, 0]$ suggests that pool excluders prevail (see Fig. 4).

8 Competition between prosocial exclusions and punishments in the compulsory PGG

For the compulsory PGG, in the absence of second-order exclusion and punishment the average payoff for cooperators is given by

$$\Pi_X = rc - c - \frac{\binom{M-E-F-1}{N-1}}{\binom{M-1}{N-1}} \frac{rc(N-1)Y}{N(M-E-F-1)}.$$

The payoff for defectors is

$$\begin{aligned} \Pi_Y = & \frac{(N-1)(-W\gamma - VB)}{M-1} - \frac{\binom{M-E-F-1}{N-1}}{\binom{M-1}{N-1}} [\frac{(N-1)(-W\gamma - VB)}{M-1} - \\ & \frac{rc(N-1)(M-E-F-Y) - N(N-1)(W\gamma + VB)}{N(M-E-F-1)}]. \end{aligned}$$

The payoff for peer excluders is

$$\Pi_E = rc - c - \frac{(N-1)Yc_E}{M-1}.$$

The payoff for peer punishers is

$$\Pi_W = rc - c - \frac{(N-1)Y\beta}{M-1} - \frac{\binom{M-E-F-1}{N-1}}{\binom{M-1}{N-1}} [\frac{rc(N-1)(M-1)Y - N(E+F)(N-1)Y\beta}{N(M-1)(M-E-F-1)}].$$

The payoff for pool excluders is

$$\Pi_F = rc - c - \delta.$$

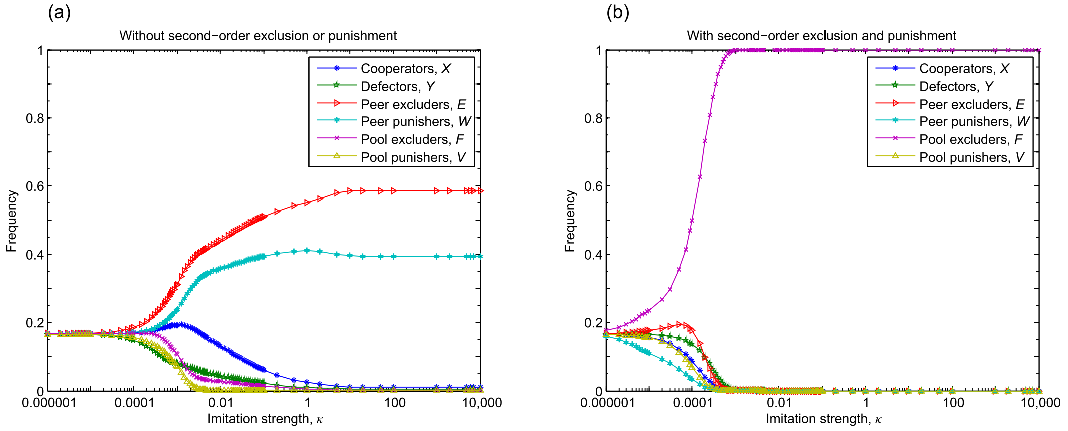

FIG. S4: (Color online) The competition among peer excluders, pool excluders, peer punishers, and pool punishers in the compulsory PGG. In the absence of second-order exclusion and punishment, peer excluders can outperform other strategies, and peer punishers can survive in the population (a). With second-order exclusion and punishment, pool excluders win (b). Parameters: $N = 5$, $M = 100$, $c = 1$, $r = 3$, $c_E = 0.4$, $\delta = 0.4$, $B = G = 0.4$, and $\beta = \gamma = 0.4$.

The payoff for pool punishers is

$$\Pi_V = rc - c - G - \frac{\binom{M-E-F-1}{N-1}}{\binom{M-1}{N-1}} \frac{rc}{N} \frac{(N-1)Y}{M-E-F-1}.$$

For small exploration rates, the transition matrix between cooperators (X), defectors (Y), peer excluders (E), peer punishers (W), pool excluders (F), and pool punishers (V) is given by

$$\begin{pmatrix} I_X & \frac{\rho_{XY}}{5} & \frac{\rho_{XE}}{5} & \frac{\rho_{XW}}{5} & \frac{\rho_{XF}}{5} & \frac{\rho_{XV}}{5} \\ \frac{\rho_{YX}}{5} & I_Y & \frac{\rho_{YE}}{5} & \frac{\rho_{YW}}{5} & \frac{\rho_{YF}}{5} & \frac{\rho_{YV}}{5} \\ \frac{\rho_{EX}}{5} & \frac{\rho_{EY}}{5} & I_E & \frac{\rho_{EW}}{5} & \frac{\rho_{EF}}{5} & \frac{\rho_{EV}}{5} \\ \frac{\rho_{WX}}{5} & \frac{\rho_{WY}}{5} & \frac{\rho_{WE}}{5} & I_W & \frac{\rho_{WF}}{5} & \frac{\rho_{WV}}{5} \\ \frac{\rho_{FX}}{5} & \frac{\rho_{FY}}{5} & \frac{\rho_{FE}}{5} & \frac{\rho_{FW}}{5} & I_F & \frac{\rho_{FV}}{5} \\ \frac{\rho_{VX}}{5} & \frac{\rho_{VY}}{5} & \frac{\rho_{VE}}{5} & \frac{\rho_{VW}}{5} & \frac{\rho_{VF}}{5} & I_V \end{pmatrix},$$

where $I_K = 1 - \sum_{K \neq L} \frac{\rho_{KL}}{5}$, and $K, L \in \{X, Y, E, W, F, V\}$. In the strong imitation limit, the

transition matrix is

$$\begin{pmatrix} \frac{4}{5}-\frac{2}{5M} & \frac{1}{5} & \frac{1}{5M} & \frac{1}{5M} & 0 & 0 \\ 0 & \frac{3}{5} & \frac{1}{5} & 0 & \frac{1}{5} & 0 \\ \frac{1}{5M} & 0 & 1-\frac{2}{5M} & \frac{1}{5M} & 0 & 0 \\ \frac{1}{5M} & 0 & \frac{1}{5M} & 1-\frac{2}{5M} & 0 & 0 \\ \frac{1}{5} & 0 & \frac{1}{5} & \frac{1}{5} & \frac{2}{5}-\frac{1}{5M} & \frac{1}{5M} \\ \frac{1}{5} & 0 & \frac{1}{5} & \frac{1}{5} & \frac{1}{5M} & \frac{2}{5}-\frac{1}{5M} \end{pmatrix}.$$

Here, the stationary distribution is $[\frac{6}{5M+22}, \frac{3}{5M+22}, \frac{3M+6}{5M+22}, \frac{2M+6}{5M+22}, \frac{3M+1}{(5M+22)(3M+2)}, \frac{1}{(5M+22)(3M+2)}]$, which shows that peer excluders has an evolutionary advantage over other strategy individuals (see Fig. S4).

If we consider the second-order exclusion and punishment, then the average payoff for cooperators is given by

$$\begin{aligned}\Pi_X \;=\; & \frac{\binom{M-E-F-1}{N-1}}{\binom{M-1}{N-1}}[\frac{(N-1)(W\gamma+VB)}{M-1)}+rc-\frac{rc(N-1)Y}{(M-E-F-1)N}-\frac{(N-1)(VB+W\gamma)}{M-E-F-1}] \\ & -c-\frac{(N-1)(VB+W\gamma)}{M-1}.\end{aligned}$$

The payoff for peer excluders is

$$\begin{aligned}\Pi_E \;=\; & [1-\frac{\binom{M-F-1}{N-1}}{\binom{M-1}{N-1}}][-c-\frac{(N-1)(X+Y+W+V)c_E+(N-1)(W\gamma+VB)}{M-1}]+\frac{\binom{M-F-1}{N-1}}{\binom{M-1}{N-1}} \\ & \sum_{i=0}^{N-1}\sum_{j=0}^{N-1-i}\sum_{p=0}^{N-1-i-j}\sum_{t=0}^{N-1-i-j-p}\frac{\binom{M-F-X-W-Y-V-1}{N-i-j-p-t-1}\binom{X}{i}\binom{Y}{j}\binom{W}{t}\binom{V}{p}}{\binom{M-F-1}{N-1}}[\frac{rc(N-j)}{N-i-j-p-t}- \\ & t\gamma-pB-(i+j+t+p)c_E-c].\end{aligned}$$

The payoff for peer punishers is

$$\begin{aligned}\Pi_W \;=\; & -c-\frac{(N-1)(X+Y+E+F)\beta+(N-1)VB}{M-1}- \\ & \frac{\binom{M-E-F-1}{N-1}}{\binom{M-1}{N-1}}\{-\frac{(N-1)(X+Y+E+F)\beta+(N-1)VB}{M-1}-rc+ \\ & \frac{rc}{N}\frac{(N-1)Y}{M-E-F-1}+\frac{(N-1)[(X+Y)\beta+VB]}{M-E-F-1}\}.\end{aligned}$$

The payoff for pool excluders is

$$\begin{aligned}\Pi_F \;=\; & \sum_{i=0}^{N-1}\sum_{j=0}^{N-i-1}\sum_{p=0}^{N-i-j-1}\sum_{t=0}^{N-i-j-p-1}\frac{\binom{M-E-X-Y-W-V-1}{N-i-j-p-t-1}\binom{X+E}{i}\binom{Y}{j}\binom{V}{p}\binom{W}{t}}{\binom{M-1}{N-1}} \\ & \times[\frac{r(N-j)c}{N-i-j-p-t}-c-\delta-t\gamma-pB].\end{aligned}$$

Last, the payoff for pool punishers is

$$\Pi_V = -c - G - \frac{\binom{M-E-F-1}{N-1}}{\binom{M-1}{N-1}}[\frac{rc}{N}\frac{(N-1)Y}{M-E-F-1} - rc].$$

In the strong imitation limit the transition matrix is given by

$$\begin{pmatrix} \frac{2}{5} & \frac{1}{5} & \frac{1}{5} & 0 & \frac{1}{5} & 0 \\ 0 & \frac{3}{5} & \frac{1}{5} & 0 & \frac{1}{5} & 0 \\ 0 & 0 & \frac{4}{5} & 0 & \frac{1}{5} & 0 \\ 0 & 0 & \frac{1}{5} & \frac{3}{5} & \frac{1}{5} & 0 \\ 0 & 0 & 0 & 0 & 1 & 0 \\ 0 & 0 & \frac{1}{5} & 0 & \frac{1}{5} & \frac{3}{5} \end{pmatrix}.$$

The resulting stationary distribution is $[0, 0, 0, 0, 1, 0]$, which suggests that pool excluders win (see Fig. S4).

9 Robustness of main findings

In the last section we present some representative examples to illustrate that our main findings are robust and remain valid in a broad range of model parameters. First, in Fig. S5 we show the result of competition between peer exclusion and peer punishment strategies in the optional PGG for a significantly larger $\beta = 0.7$ fine value. Note that here the fine is almost two times higher than the punishment cost. Still, excluder strategy remains dominant in the absence of second-order sanctioning for almost all imitation strength values. If the second-order punishment and exclusion are possible then peer exclusion prevails again, as shown in the right panel of Fig. S5.

In agreement with our previous findings the superiority of peer exclusion remains intact no matter how the fine is increased relevantly in the presence of pool strategies. A typical outcome is shown in Fig. S6 where in most of the time the majority of individuals prefer peer exclusion even if pool strategies are possible in the absence of second order sanctioning. If the latter is possible then pool exclusion prevails, as it is demonstrated in the right panel of Fig. S6.

Our last figure illustrates the frequencies of available strategies by choosing different group sizes for the public goods game. Both panels of Fig. S7 highlight that group size has no significant role in the competition of strategies no matter whether second-order sanctioning is considered or not.

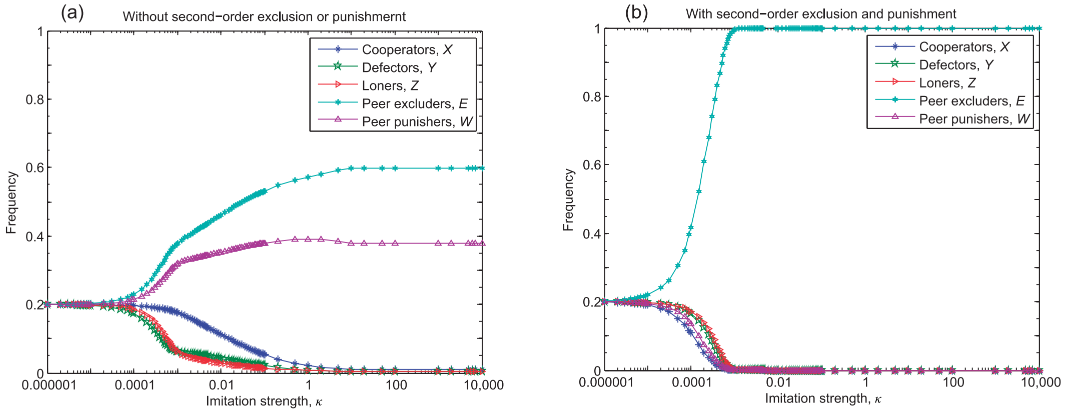

FIG. S5: (Color online) The competition between peer exclusion and peer punishment in the optional PGG for a larger punishment fine $\beta = 0.7$. In the absence of second-order sanctioning, shown in panel (a), both strategies survive but peer exclusion dominates. If second-order exclusion and punishment are applied then peer excluders prevail, as shown in panel (b). Parameters: $N = 5$, $r = 3$, $c = 1$, $\mu = 10^{-6}$, $\sigma = 1$, $M = 100$, $c_E = 0.4$, and $\gamma = 0.4$.

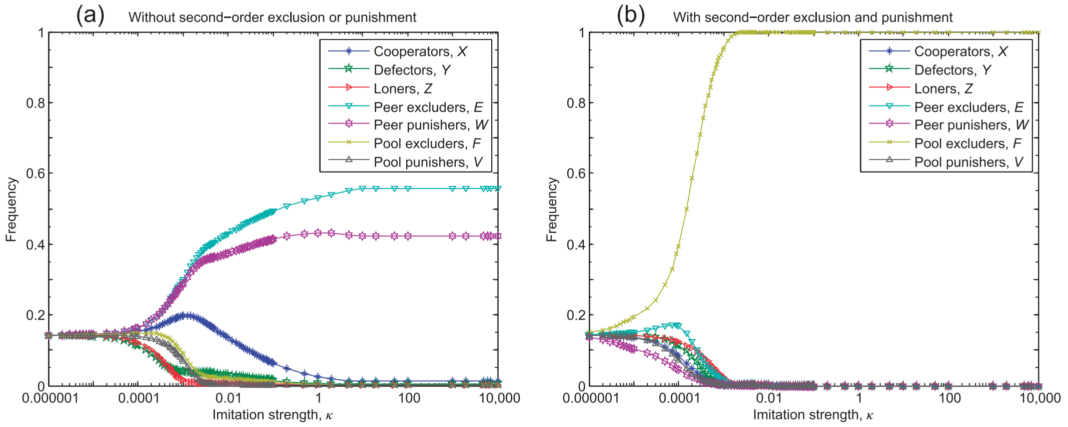

FIG. S6: (Color online) The competition between different types of social exclusion and costly punishment in the optional PGG for a large punishment fine $\beta = 0.7$. When strategies can penalize defectors only, shown in panel (a), then all strategies coexist in time average for weak strength of imitation, but in most of the time peer excluders form the majority of the population for other imitation strength values. Panel (b) shows the case when second-order sanctioning is present. Here pool excluders prevail and dominate the whole population. Parameters: $N = 5$, $r = 3$, $c = 1$, $\mu = 10^{-6}$, $\sigma = 1$, $M = 100$, $c_E = \delta = 0.4$, $\gamma = 0.4$, and $B = G = 0.4$.

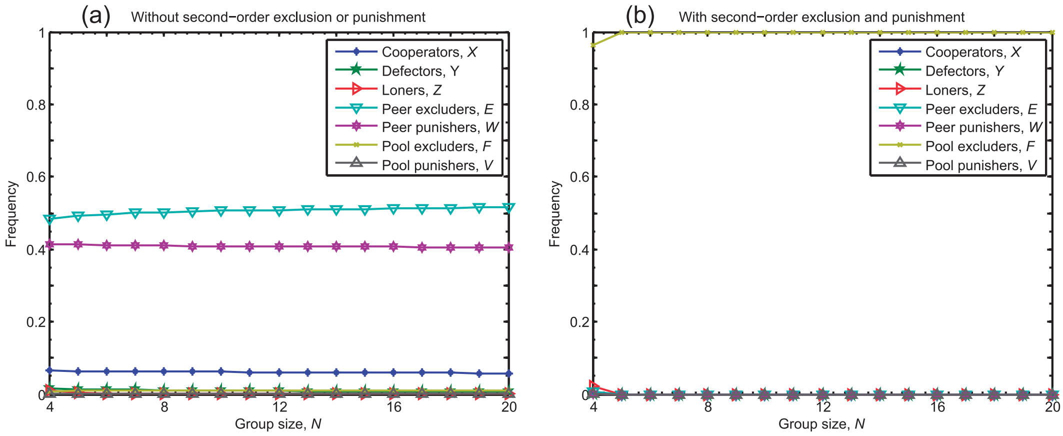

FIG. S7: (Color online) The competition between different types of social exclusion and costly punishment in the optional PGG as a function of the group size N for imitation strength $\kappa = 0.1$. It is shown that group size has no significant role in the competition of strategies no matter whether second-order sanctioning is considered or not. Parameters: $r/N = 0.6$, $c = 1$, $\mu = 10^{-6}$, $\sigma = 1$, $M = 100$, $c_E = \delta = 0.4$, $\beta = \gamma = 0.4$, and $B = G = 0.4$.

---

[1] Abdallah, S., Sayed, R., Rahwan, I., LeVeck, B. L. & Cebrian, M. Corruption drives the emergence of civil society. *J. R. Soc. Interface* **11**, 20131044 (2014).

[2] Hauert, C., Traulsen, A., Brandt, H., Nowak, M. A. & Sigmund, K. Via freedom to coercion: the emergence of costly punishment. *Science* **316**, 1905-1907 (2007).

[3] Sigmund, K., De Silva, H., Traulsen, A. & Hauert, C. Social learning promotes institutions for governing the commons. *Nature* **466**, 861-863 (2010).

[4] Traulsen, A., Nowak, M. A. & Pacheco, J. M. Stochastic dynamics of invasion and fixation. *Phys. Rev. E* **74**, 011909 (2006).
